# Multi-Omics Reveals Active Components and Mechanisms of Heat-Processed Gypenosides Hepatoprotective Against APAP Injury

**DOI:** 10.3390/biom15111555

**Published:** 2025-11-05

**Authors:** Peng Xie, Qiuru Li, Shu Jiang, Miao Sun, Yu Duan, Changping Hu, Xianglan Piao

**Affiliations:** 1 Shanxi Provincial Department-Municipal Key Laboratory Cultivation Base for Quality Enhancement and Utilization of Shangdang Chinese Medicinal Materials, School of Pharmacy, Changzhi Medical College, Changzhi 046000, Shanxi, Chinashujiang@czmc.edu.cn (S.J.);; 2 China Academy of Traditional Chinese Medicine, Beijing 100081, China; 3 School of Pharmacy, Minzu University of China, Beijing 100081, China

**Keywords:** *Gynostemma pentaphyllum*, gypenosides, liver injury, multiomics, molecular docking, underlying mechanism

## Abstract

This study elucidates the hepatoprotective mechanisms of heat-processed *Gynostemma pentaphyllum* (Thunb.) Makino saponins (HGyp) against APAP-induced liver injury using serum pharmacochemistry, metabolomics, and network pharmacology. HGyp significantly mitigated liver damage in mice, as confirmed by biochemical and histopathological analyses. UPLC-MS identified 38 bioactive compounds, including 16 prototype saponins and 11 metabolites. Network pharmacology and molecular docking revealed damulin A/B, gypenosides (L/LI/LVI/XLVI), and ginsenosides (Rg3/Rd) as key components targeting GRB2, FGF2, MMP2, STAT3, CASP3, and HSP90A. Western blotting confirmed the HGyp-mediated downregulation of hepatic HSP90A and STAT3. Metabolomics identified four critical pathways, PPAR, ferroptosis, and the inflammatory mediator regulation of TRP channels involved in hepatoprotection. HGyp exerts multi-target effects via anti-inflammatory activity, apoptosis, and metabolism, providing a framework for Chinese medicine and ethnomedicine research.

## 1. Introduction

Liver injury (LI) represents a multifactorial disorder marked by key pathological manifestations such as parenchymal cell death, immune cell recruitment, and compromised liver function. It has diverse clinical manifestations such as fatigue and anorexia. The most prominent hallmark is a significant elevation of serum transaminases [1,2]. Without timely intervention, LI may progress to cirrhosis, liver failure, or even hepatocellular carcinoma [3,4]. Converging lines of evidence indicate that the pathogenesis of LI involves multifactorial interactions, encompassing mitochondrial dysfunction, oxidative stress burst, the hyperactivation of inflammatory signaling pathways, imbalance between apoptosis/necroptosis, and hepatic stellate cell abnormalities [5,6]. While N-acetylcysteine (NAC) has FDA approval for the clinical management of acute hepatic injury, its efficacy in chronic liver injury remains limited [7]. Conversely, the clinical application of glucocorticoids is constrained by severe adverse effects [8]. Acetaminophen (APAP) hepatotoxicity represents the predominant etiology of medication-related hepatic damage and fulminant liver failure across Western populations, comprising approximately half of ALF cases in North America (46%) and a majority in European nations (40–70%) [9]. However, the current standard treatment, NAC, has limitations, including a narrow therapeutic window (effective only within 8 h post-overdose) and suboptimal efficacy in advanced liver injury. Emerging therapeutic strategies primarily target oxidative stress modulation [9], anti-inflammatory pathways [10], and gene therapy [11]. Despite considerable advances in hepatoprotective drug discovery, most drug candidates remain in clinical development. This underscores the necessity of adopting multi-target intervention strategies to address the complex pathogenesis of acute liver injury.

The contemporary advancement of Traditional Chinese Medicine (TCM) and ethnopharmacology encounters a fundamental bottleneck: the scientific elucidation of their complex chemical composition systems and holistic mechanisms of action [12]. In recent years, multidisciplinary technologies have provided innovative solutions to overcome this bottleneck. Serum pharmacochemistry analyzes blood-absorbed components and their metabolites, accurately identifying the direct bioactive substances of TCM in vivo, thereby effectively avoiding the false-positive/negative results associated with traditional in vitro isolation methods [13,14]. Network pharmacology constructs a multidimensional “components–targets–pathways–diseases” network to systematically decipher the integrated regulatory mechanisms underlying the synergistic effects of multiple components on multiple targets [15,16]. Metabolomics monitors dynamic changes in endogenous metabolites during disease states, not only providing objective indicators for evaluating TCM efficacy but also revealing its metabolic regulation essence [17]. The integrated research framework combining “bioavailable components–metabolic regulation–network targets” [18] not only fully preserves the holistic concept and syndrome differentiation characteristics of TCM, but also employs modern scientific approaches to elucidate the multi-component, multi-target mechanisms of herbal medicines.

*Gynostemma pentaphyllum* (Thunb.) Makino (the plant name has been checked with MPNS (http://mpns.kew.org), accessed on 25 July 2025) has demonstrated remarkable pharmacological activity in liver disease treatment with favorable safety profiles [19,20]. As a TCM and Zhuang ethnomedicine, its major bioactive components include gypenosides, flavonoids, and polysaccharides, which exhibit antioxidant, anti-inflammatory, and hepatoprotective effects [21,22]. First documented in “Jiu huang ben cao”, and in “Compendium of Materia Medica”, it has been used in China for centuries to improve liver function, alleviate hepatic injury, and regulate metabolic disorders. Traditional medicine documents *G. pentaphyllum*. for detoxification and liver diseases, i.e., hepatoprotective activities [21,22]. Modern clinical studies further confirm that its total saponins exhibit lipid-lowering and liver-protective effects [19,23]. Recent studies indicate that *G. pentaphyllum* and its extracts show promising therapeutic effects in disease models including metabolic-associated fatty liver disease (MAFLD), hepatic fibrosis, and liver injury [24,25,26,27]. Preliminary research revealed that heat-processed methods significantly alter the composition and content of gypenosides, with demonstrated efficacy against lipid accumulation disorder [24,28]. Nevertheless, the active components responsible for its hepatoprotective effects after heat processing and their underlying molecular mechanisms remain incompletely understood and require further investigation.

**Figure 1:**
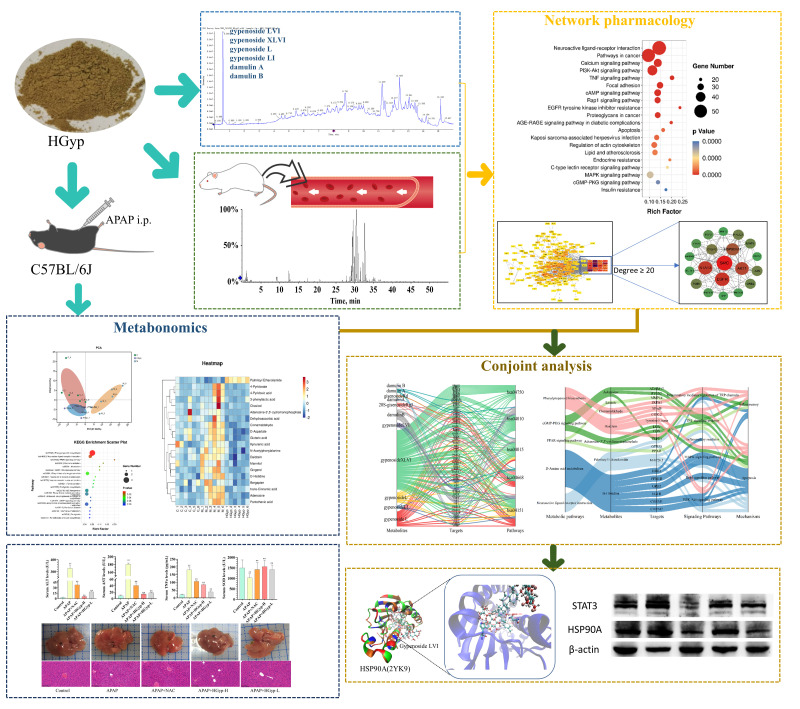
Abstract of this study.

This study employed a multi-omics integration strategy to systematically elucidate, for the first time, the anti-hepatotoxic material basis and hepatoprotective mechanisms of HGyp. First, UPLC-MS was utilized to characterize the blood-absorbed components of the heat-processed *G. pentaphyllum* extract. The hepatoprotective efficacy of HGyp was then evaluated using an APAP hepatotoxicity model. Subsequently, network pharmacology was applied to construct a multidimensional “components–targets–pathways” interaction network to identify core bioactive components and key targets. Further, metabolomics analysis was performed to explore HGyp regulated metabolic pathways. Molecular docking and Western blotting were employed to validate HGyp’s effects on core targets. This study not only reveals the scientific connotation of HGyp’s active components and mechanisms, but also provides a method for the modernization research of ethnomedicine (Figure 1).

## 2. Materials and Methods

### 2.1. Materials

*G. pentaphyllum* was purchased in Zhangzhou, Fujian, China, and was professionally identified. The voucher specimen (No. GP 2016-01) was placed in the Isolation and Structure Identification Laboratory in Minzu University of China. Hematoxylin and eosin (H&E) dye was used (Wuhan Servicebio Biotechnology Co., Ltd., Wuhan, China). Acetaminophen (APAP, purity > 98%) and N-acetyl-L-cysteine (NAC, purity > 98%) were purchased from Macklin Biochemical Technology Co., Ltd. (Shanghai, China). Alanine aminotransferase (ALT), aspartate aminotransferase (AST), superoxide dismutase (SOD), and TNF-α ELISA assay kits were purchased from the Nanjing Jiancheng Bioengineering Institute (Nanjing, China). A BCA protein assay kit was obtained from Beijing LABLEAD Technology Co., Ltd. (Beijing, China). Antibodies against β-actin, STAT3, and HSP90 were procured from Bioss Biotechnology Co., Ltd. (Beijing, China).

### 2.2. Extract Preparation, Quantification and Component Analysis of HGyp

Dried *G. pentaphyllum* leaves (1.0 kg) were heat-processed at 120 °C under 0.24 MPa pressure for 3 h. The processed material was then extracted three times with 80% ethanol (3 h each) under reflux conditions. The combined extracts were concentrated to obtain a crude extract, which was lyophilized to yield the total ethanol extract. For further purification, HP20 macroporous adsorption resin was employed as the stationary phase, with sequential elution using water, 50% ethanol, and 95% ethanol. The 95% ethanol-eluted fraction was concentrated to obtain HGyp, which was subsequently lyophilized for storage and future use.

An accurately weighed quantity of the gypenoside LVI reference standard was dissolved in ethanol to prepare a 1 mg/mL reference standard solution. HGyp was precisely weighed and dissolved in ethanol to prepare a 1 mg/mL sample solution. Then, 0.5 mL of the test sample solution and 0.5 mL of the gypenoside LVI solution were separately transferred into test tubes. To each tube, 0.5 mL of vanillin–ethanol solution and 0.5 mL of ethanol (99.7% *v*/*v*) were added, followed by the addition of 5 mL of 72% sulfuric acid. The mixtures were incubated in a water bath at 60 °C for 10 min and then immediately cooled in an ice-water bath for 2 h. The absorbance of the resulting solutions was measured at the maximum absorption wavelength of 550 nm. The total saponin content was calculated based on the established standard curve.

The qualitative analysis of HGyp was performed using a Thermo UPLC system (Thermo, Waltham, MA, USA) equipped with a BEH C18 column (100 mm × 2.1 mm, 1.7 μm; Waters, Milford, MA, USA). The mobile phase consisted of 0.1% formic acid in water (A) and acetonitrile (B) with the following gradient program: 0–3.5 min, 2–25% B; 3.5–7.5 min, 25–35% B; 7.5–11 min, 35–50% B; and 11–13 min, 50–95% B. The flow rate was maintained at 0.4 mL/min with an injection volume of 5 μL. Compound identification was achieved by referencing both our research team’s prior expertise in the chemical characterization of heat-processed *G. pentaphyllum* and established analytical results [24,28,29,30,31,32,33].

### 2.3. Animal Experiments

We procured 6–8-week-old male C57BL/6 mice (18–22 g) from Beijing Sibeifu Laboratory Animal Technology Co., Ltd., Beijing, China. The animals were maintained in SPF conditions with the following environmental parameters: ambient temperature: 23 ± 2 °C, relative humidity: 50 ± 5%, and photoperiod: 12 h circadian lighting. Following a 7-day acclimation period with daily health monitoring, mice were stratified by weight and randomly allocated to five experimental cohorts (*n* = 10/group) using computer-generated randomization: (1) the control group, (2) APAP (400 mg/kg) model group, (3) APAP (400 mg/kg) + HGyp-H (200 mg/kg) group, (4) APAP (400 mg/kg) + HGyp-L (100 mg/kg) group, and (5) APAP (400 mg/kg) + NAC (150 mg/kg) positive drug control group. The doses of HGyp (100 and 200 mg/kg) were selected based on their previously demonstrated efficacy in metabolic disease models and a conversion from the traditional clinical dosage (15–30 g of crude herb) used in Traditional Chinese Medicine [19,24]. The dose of NAC (150 mg/kg) was chosen according to established protocols for APAP-induced liver injury [34]. All treatments were administered via oral gavage once daily for 7 days. The blank control and APAP groups received an equivalent volume of carboxymethyl cellulose sodium (CMC-Na) solution. All mice were administered at a dose of 0.2 mL/10 g body weight. HGyp and NAC were dissolved in 2% CMC-Na solution, while APAP was dissolved in normal saline. Except for the control group, all animals received an i.p. injection of 400 mg/kg APAP solution 2 h after the last administration. The control group received an equal volume of normal saline (i.p.). Blood and liver samples were collected 24 h after APAP injection. Figure 3A illustrates the experimental design of the in vivo study. All animal experiments were approved by the Laboratory Animal Ethics Committee of Changzhi Medical College (Approval No. DW2025046).

### 2.4. Serum Biochemical Assays, Organ Index, and Histopathological Analysis of Liver Damage

At the termination of the experiment, major organs (the heart, liver, spleen, lungs, and kidneys) were carefully excised and precisely weighed from each mouse. Organ index (%) = (M_or_g_an_/M_mouse_) × 100%, M_mouse_ represents the body weight (g) and M_or_g_an_ denotes the organ weight (g). Following centrifugation (12,000× *g*, 15 min, 4 °C), plasma aliquots were analyzed for hepatic injury markers (AST and ALT) and oxidative stress/inflammatory parameters (SOD and TNF-α) using validated commercial kits, with strict adherence to kit specifications. Upon collection, the left hepatic lobe was immersion-fixed in 4% paraformaldehyde for 48 h under ambient conditions. Following fixation, tissues were dehydrated, cleared, and embedded in paraffin blocks. Serial sections (5 μm) were prepared using a rotary microtome and subjected to conventional H&E staining for morphological evaluation. The histopathological scoring of hepatic damage was performed by light microscopy, with grading based on characteristic architectural alterations.

### 2.5. Metabolomics

#### 2.5.1. Sample Preparation

Aliquots of serum (100 μL) were transferred to 2.0 mL polypropylene microtubes and mixed with 400 μL of pre-chilled methanol (−20 °C). The solution was homogenized by vortex mixing (3 × 30 sec pulses) to achieve complete protein denaturation. Following incubation at −20 °C for 30 min to enhance precipitation, phase separation was accomplished by high-speed centrifugation (20,000× *g*, 15 min, 4 °C). The clarified supernatant (400 μL) was aspirated, filtered through a 0.22 μm nylon membrane, and centrifuged again under identical parameters to remove residual particulates. The final extract was stored in certified pre-cleared LC-MS vials prior to UPLC-HRMS profiling. Equal aliquots (20 μL) from all sample extracts were pooled to generate a composite QC sample, which was analyzed intermittently throughout the analytical sequence. All procedures were performed on ice. This sample preparation method is optimized for broad coverage of polar and moderately lipophilic metabolites [35].

#### 2.5.2. Experimental Method

Chromatographic separation was conducted on a Waters ACQUITY UPLC I-Class platform (Waters, Milford, MA, USA) employing an ACQUITY UPLC T3 C18 column (100 × 2.1 mm, 1.8 μm; Waters, Wilmslow, UK) maintained at 40 °C with a constant flow rate of 0.3 mL/min. The binary mobile phase system consisted of (A) an aqueous solution containing 5 mM ammonium acetate and 5 mM acetic acid and (B) neat acetonitrile. A multi-step gradient profile was implemented: 0–2 min, 5–70% B and 2–6 min, 70–99% B. Mass spectrometric detection was carried out using a Q-Exactive Plus Orbitrap mass spectrometer (Thermo Scientific, Bremen, Germany) configured with a heated electrospray ionization source operating at 350 °C capillary temperature. Optimized ionization parameters included sheath and auxiliary gas flows of 30 and 10 arbitrary units, respectively, with spray voltages of +4.0 kV (positive ion mode) or −2.8 kV (negative ion mode). Full-scan mass spectra (*m*/*z* 70–1050) were acquired at 70,000 resolution (*m*/*z* 200) with a 100 ms maximum injection time, while data-dependent MS/MS acquisition automatically triggered the fragmentation of the five most abundant precursor ions (intensity threshold > 1 × 10^5^ counts) at 17,500 resolution using a 50 ms maximum injection time. To ensure data quality, pooled quality control samples were analyzed after every three experimental injections, enabling both system performance monitoring and post-acquisition mass accuracy calibration through inter-batch QC alignment.

#### 2.5.3. Data Analysis

The untargeted metabolomics data analysis was performed through XCMS Online platform (Scripps Research Institute) for comprehensive peak extraction, retention time adjustment, and intensity normalization to ensure data comparability. To explore metabolic patterns and discriminate sample groups, we employed advanced chemometric approaches comprising unsupervised PCA modeling and supervised OPLS-DA regression were performed using R4.3.2 software. Heatmaps were generated to visualize inter-group variations and clustering patterns among samples. Biomarker selection criteria included statistical significance (*p* < 0.05), fold change ≥ 1.2, and VIP scores >1.0. Tentative metabolite identification was achieved by matching against the Human Metabolome Database (HMDB). Metabolic pathway analysis was conducted using GSEA (v4.1.0). Significantly enriched pathways were filtered using the following thresholds: |NES| > 1, NOM *p* < 0.05, and FDR q < 0.25.

### 2.6. Analysis of Migrating Components in Blood

#### 2.6.1. Administration of Drugs to Animals and Sample Collection

Six male Sprague-Dawley rats (170–200 g, SPF) were acclimatized in a temperature- and humidity-controlled vivarium (23 ± 2 °C, 50 ± 5% RH, 12 h photocycle) with ad libitum access to food and water, following their procurement from Beijing Sibeifu Biotechnology. Following a 7-day environmental adaptation period, the heat-processed *G. pentaphyllum* ethanol extract was suspended in 2% CMC-Na solution (300 mg/mL suspension) for oral administration. Prior to the experiment, all rats were fasted for 12 h with free access to water. Baseline blood samples were collected via the orbital vein before drug administration. Animals received the test extract via oral gavage (2 mL/100 g BW). Retro-orbital blood collection (0.5 mL aliquots) was performed at predetermined intervals (0 min, 5 min, 30 min, 45 min, 1 h, 1.5 h, 4 h, 8 h, 12 h, and 24 h post-administration) using heparinized microtainers. Following 30 min coagulation at ambient temperature, the samples were processed by refrigerated centrifugation (3500 rpm, 10 min, 4 °C), with harvested plasma immediately frozen at −80 °C in cryovials for batch analysis.

#### 2.6.2. Sample Preparation and Detection

Aliquots (100 μL) of plasma collected at each time point were mixed with 300 μL of acetonitrile (1:3, *v*/*v*) and vortexed vigorously. Following high-speed centrifugation (12,000 rpm, 15 min, 4 °C), the clarified supernatant was concentrated to complete dryness using nitrogen gas evaporation. The dried extracts were then redissolved in methanol with vigorous vortex mixing (5 min) to ensure full solubilization, followed by repeat centrifugation (12,000 rpm, 15 min, 4 °C) to remove particulates. The final processed samples were transferred to autosampler vials for LC-MS analysis.

Chromatographic separation was accomplished on a reversed-phase Acquity UPLC BEH C18 column (50 × 2.1 mm, 1.7 μm; Waters) at 30 °C, with a mobile phase system of (A) aqueous 0.1% formic acid and (B) acetonitrile delivered at 0.3 mL/min. Samples (5 μL) were injected for mass spectrometric analysis using Q-TOF detection. The gradient elution program: 0–10 min, 12–25% B; 10–25 min, 25–40% B; 25–33 min, 40–70% B; 33–40 min, and 70–95% B. Mass spectrometric detection was conducted in negative ion mode with ESI: CUR, 40 psi; CAD, medium; IS, −4500 V; TEM, 550 °C; GS1, 55 psi; and GS2, 55 psi. IDA mode with full-scan MS (*m*/*z* 200–1500; DP, −80 V; CE, −10 V) and MS/MS scans (*m*/*z* 50–1500; DP, −80 V) was implemented, selecting the top 8 most intense ions for fragmentation.

The ethanol-soluble components of heat-processed *G. pentaphyllum* were characterized through cross-referencing with five major chemical databases (ChemSpider, http://www.chemspider.com (accessed on 20 June 2025); PubChem, http://pubchem.ncbi.nlm.nih.gov (accessed on 20 June 2025); ChemBank, http://chembank.med.harvard.edu (accessed on 20 June 2025); MassBank, http://www.massbank.jp (accessed on 20 June 2025); and ScienceDirect). The acquired database records were cross-referenced with observed MS/MS fragmentation patterns and the existing literature to identify potential structures of the blood-absorbed components, encompassing both prototype saponins and their metabolites derived from the heat-processed *G. pentaphyllum* extract.

### 2.7. Network Pharmacology

#### 2.7.1. Prediction and Intersection of Targets

To elucidate the potential therapeutic targets of HGyp against liver injury, we employed a systematic approach combining an analysis of blood-absorbed components and prototype constituents from HGyp as potential active ingredients. Compound–target interactions were predicted through parallel screening using PharmMapper (http://www.lilab-ecust.cn/pharmmapper/, accessed on 20 June 2025) and SwissTargetPrediction (http://www.swisstargetprediction.ch/, accessed on 20 June 2025). Liver pathology-associated targets were systematically compiled from disease databases (GeneCards, https://www.genecards.org/, accessed on 20 June 2025, DrugBank, https://www.drugbank.ca, accessed on 20 June 2025, OMIM, https://omim.org/, accessed on 20 June 2025) through boolean searches of hepatotoxicity-related terms. The Venny 2.1 tool identified consensus targets between the compound predictions and liver injury datasets, establishing HGyp’s putative therapeutic targets for hepatic protection.

#### 2.7.2. PPI Network Construction

Consensus targets underwent PPI network construction via the STRING database (https://string-db.org/, accessed on 20 June 2025) followed by visualization in Cytoscape (v3.10.1). Topological analysis employing CytoNCA plugin evaluated four centrality metrics (betweenness, closeness, degree, and local average connectivity), with hub targets identified as nodes exceeding median values across all parameters.

#### 2.7.3. GO and KEGG Enrichment Analysis

Consensus targets underwent comprehensive functional annotation through DAVID (https://david.ncifcrf.gov/, accessed on 20 June 2025), analyzing three GO domains (biological processes, BP; cellular components, CC; and molecular functions, MF) and KEGG pathways. Statistically significant associations (*p* < 0.05) were identified and visualized to highlight key mechanistic pathways relevant to hepatoprotection.

#### 2.7.4. “Compound–Target–Pathway” Network Construction

To establish the comprehensive C-T-P network, we performed topological parameter analysis using Cytoscape. Based on network centrality evaluation, potential core targets were identified by applying a degree threshold > 20. The top network-identified targets (based on degree centrality) were computationally screened against HGyp phytochemicals through molecular docking to assess binding potential.

### 2.8. Molecular Docking

Following energy minimization using Chem3D (ChemDraw12.0) software, the bioactive components were converted into mol_2_ format for subsequent docking studies. Seven target protein structures, CASP3 (PDB:1NME), HSP90 (PDB:2YK9), FGF2 (PDB:4OEF), STAT3 (PDB:6NJS), AKT1 (PDB:1UNQ), MMP2 (PDB:8H78), and GRB2 (PDB:6ICG), were retrieved from the PDB database. Protein structures were processed by removing non-essential organic molecules and solvent molecules. The protein structures were prepared using GLOD 5.0 by removing solvent molecules and protonating ionizable groups, enabling flexible ligand–receptor docking simulations with HGyp’s bioactive constituents. The highest-scoring docking conformations were selected for detailed visualization and analysis using Visual Molecular Dynamics (VMD) 1.9.4 software.

### 2.9. Integration of Network Pharmacology and Metabolomics

Overlapping targets identified between compound-related and pathway-related targets were used to construct a comprehensive metabolite–target–pathway network using the RAWGraphs 2.0 platform (https://app.rawgraphs.io/, accessed on 20 June 2025). The visualization integrated multiple relationship dimensions, including metabolic pathways, bioactive metabolites, validated overlapping targets (connecting both metabolites and signaling pathways), and key pathological mechanisms. This integrative analysis enabled the systematic elucidation of the potential therapeutic effects at the network pharmacology level.

### 2.10. Western Blot Assay

Liver homogenates were prepared in RIPA buffer containing a PMSF protease inhibitor, centrifuged (12,000 rpm, 15 min, 4 °C), and supernatants were quantified via a BCA assay. Protein lysates were denatured, separated by 10% SDS-PAGE, and electrotransferred to PVDF membranes. After blocking with 5% non-fat milk, membranes were probed overnight at 4 °C with primary antibodies against HSP90 (1:3000), STAT3 (1:1000), and β-actin (1:1000) as the loading control. Following TBST washes, HRP-conjugated secondary antibodies (1:5000) were applied for 1 h at RT. Protein signals were detected by ECL and quantified using ImageJ 1.53k after β-actin normalization.

### 2.11. Statistical Analysis

Quantitative data were analyzed using SPSS (version 22.0) and expressed as mean ± SD. Student’s *t*-test evaluated pairwise comparisons, while one-way ANOVA with Fisher’s LSD post hoc test assessed multi-group differences, with *p* < 0.05 considered statistically significant.

## 3. Results

### 3.1. Determination of Compounds of HGyp by LC-MS

Figure S1 displays the absorption spectrum of the gypenoside LVI reference standard solution. The results indicated a maximum absorption at a wavelength of 550 nm. A standard curve was constructed by plotting absorbance (A) on the y-axis against the content (mg) of gypenoside LVI on the x-axis. As shown in Figure S2, the regression equation for the determination of total saponins from *G. pentaphyllum* was y = 1.4157x + 0.0548, with a coefficient of determination (R^2^) of 0.9999. The results demonstrate that the total saponins (calculated as gypenoside LVI) exhibited a good linear relationship within the range of 0.04 to 0.3 mg. Based on the measurements and calculations, the total saponin content in HGyp was determined to be 747.92 mg/g.

**Figure 2:**
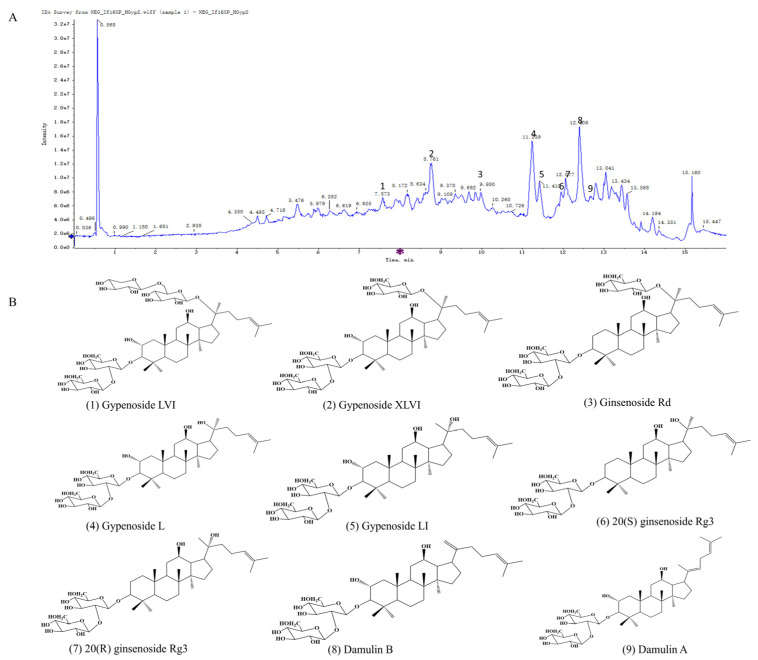
Extracted ion chromatogram of HGyp (**A**). Structures of major chemical constituents (**B**).

Figure 2A displays the total ion chromatogram (TIC) of HGyp in negative ion mode. As systematically characterized in our previous study [24,28,29,30,31,33], the major saponin constituents in HGyp were identified as gypenoside LVI, gypenoside XLVI, gypenoside L, gypenoside LI, ginsenoside Rd, 20(S)-ginsenoside Rg3, 20(R)-ginsenoside Rg3, damulin A, and damulin B (Figure 2B and Figure S3 and Table S1). According to the method previously established by our group for the simultaneous quantification of multiple components in heat-processed *G. pentaphyllum*, the contents of the major saponins in HGyp were determined as follows: ginsenoside Rd (3.782 mg/g), gypenoside LVI (5.978 mg/g), gypenoside XLVI (17.89 mg/g), gypenoside L (11.46 mg/g), gypenoside LI (5.139 mg/g), damulin A (3.269 mg/g), damulin B (4.939 mg/g), and 20(S)-ginsenoside Rg3 (0.1648 mg/g) [24,28].

### 3.2. Effect of HGyp Administration on Body Weight, Organs Weight and Index

Effects of HGyp on the weights of mice’s body, heart, liver, spleen, lungs and kidneys are shown Table 1. Compared with the control group, the APAP group exhibited marked increases in body weight (*p* < 0.05), liver weight (*p* < 0.05), and spleen weight (*p* < 0.05). liver and spleen weight were significantly attenuated by both HGyp-H and HGyp-L groups (*p* < 0.01 vs. APAP group). A comparative assessment of vital organ masses revealed no significant intergroup variations in cardiac, pulmonary, or renal tissues across the experimental cohorts.

**Figure 3:**
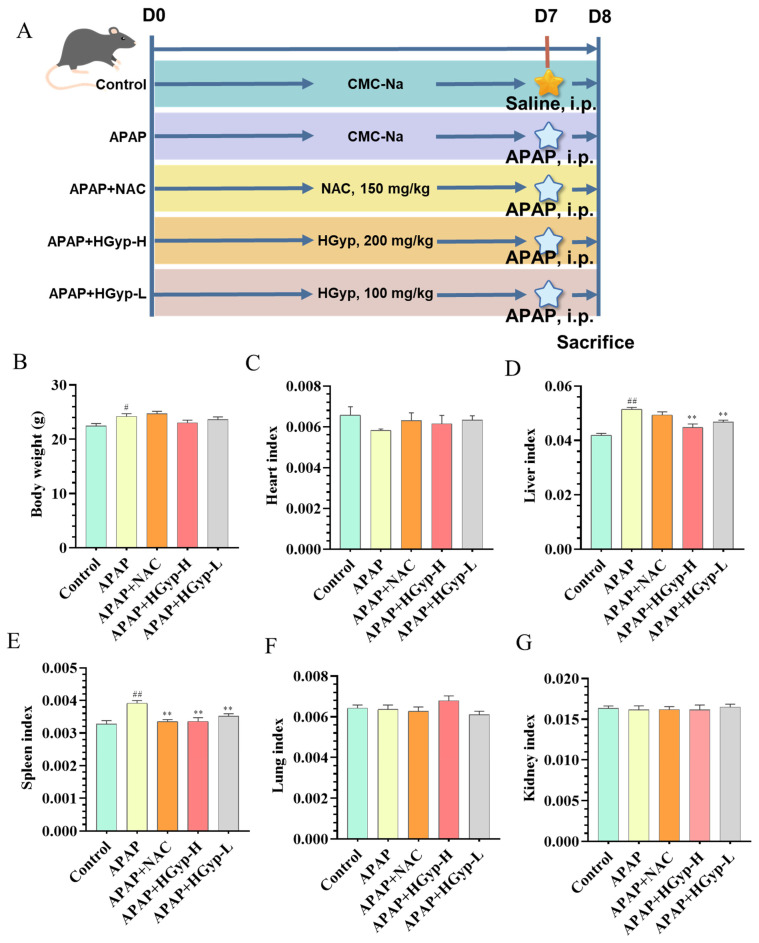
Experimental effects of HGyp on mice with APAP-induced liver injury. Mouse experimental design (**A**). Body weight (**B**). Heart index (**C**). Liver index (**D**). Spleen index (**E**). Lung index (**F**). Kidney index (**G**). Data are expressed as mean ± SD for n = 10. Control vs. APAP group ^#^ *p* < 0.05, ^##^ *p* < 0.01. HGyp vs. APAP group * *p* < 0.05, ** *p* < 0.01.

The corresponding alterations in organ indices are displayed in Figure 3B–G. The APAP group showed a significantly elevated liver index (*p* < 0.01) and spleen index (*p* < 0.01) compared to the controls, which were effectively normalized by HGyp treatment (*p* < 0.01 vs. the APAP group). The cardiac, pulmonary, and renal mass indices remained comparable across all treatment groups without statistical significance. Overall, HGyp had a relatively minor impact on the body weight and the organs of the heart, lung and kidney in the mice.

### 3.3. Effect of HGyp Administration on Serum Biochemistry and Hepatic Steatosis

**Figure 4:**
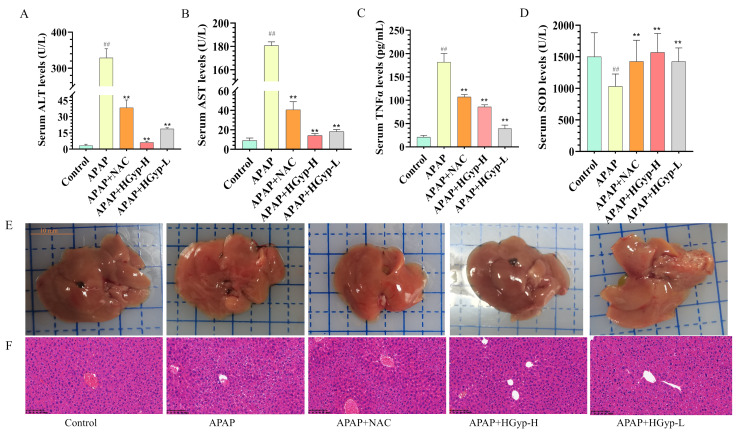
Hepatoprotective effect of HGyp against liver injury induced by APAP in mice. Serum levels of ALT (**A**), AST (**B**), TNF-α (**C**), and SOD (**D**). Liver appearance (**E**) and HE staining (**F**). Data are shown as mean ± SD for *n* = 10. ^#^ *p* < 0.05, ^##^ *p* < 0.01 vs. control group. * *p* < 0.05, ** *p* < 0.01 vs. APAP group.

Hepatic injury and inflammation are key markers for diagnosing liver damage [36]. As shown in Figure 4A–C, the APAP model group exhibited significantly higher levels of ALT, AST, and TNFα compared to the control group (*p* < 0.01), indicating hepatotoxicity and successful model establishment. In contrast, the HGyp group demonstrated a significant reduction in these markers relative to the APAP group (*p* < 0.01). As shown in Figure 4D, hepatic SOD activity was significantly suppressed in APAP-treated mice compared to the controls (*p* < 0.01), while HGyp administration effectively restored SOD levels (*p* < 0.01 vs. the APAP group). The reference drug demonstrated comparable efficacy to HGyp in normalizing ALT, AST, TNF-α, and SOD parameters.

The liver tissue morphology (Figure 4E) and histopathology (Figure 4F) provided a more intuitive assessment of hepatic injury severity than serum biochemical markers. The APAP group exhibited generalized liver swelling with scattered grayish-white or grayish-yellow patches of indistinct margins in the parenchyma, whereas the HGyp group showed improved gross morphological features compared to the APAP group. H&E staining revealed no morphological abnormalities in the control group, with intact hepatic lobule architecture and well-arranged hepatocytes. In contrast, the APAP group displayed disrupted hepatic architecture characterized by disorganized hepatocyte arrays, proliferated bile ducts, congested sinusoids, leukocyte infiltration, interstitial edema, pyknotic nuclei, dilated intercellular gaps, and multifocal necrotic lesions. But HGyp treatment partially ameliorated these APAP-induced histopathological alterations. Compared to the APAP group, HGyp-treated mice showed only mild bile duct epithelial injury, hepatocellular hydropic degeneration, and reduced neutrophil infiltration. Collectively, these results demonstrate that HGyp treatment significantly attenuates liver injury in mice.

### 3.4. Identification of Migrant Compounds of G. pentaphyllum Ethanol Extract

UPLC-Q-TOF-MS-based serum pharmacochemical analysis identified all systemic circulating components (both parent compounds and biotransformation products) as putative bioactive candidates through the comparative profiling of medicated versus blank serum samples. The total ion chromatogram is shown in Figure S4. Compounds were identified by matching retention times, accurate molecular ions, and characteristic product ions (*m*/*z* values) against an in-house database. A total of 38 compounds were detected in the post-administration rat serum, comprising 16 prototype saponins and 11 saponin metabolites. Supplementary Table S2 documents all identifiy phytochemicals, with predicted biotransformations including oxidative, sulfated, and glucuronidated metabolites, reflecting the major metabolic pathways of processed *G. pentaphyllum* constituents.

### 3.5. Metabolomics Analysis

#### 3.5.1. Metabolic Profile Analysis

To characterize metabolic disparities between experimental groups, we conducted complementary unsupervised PCA and supervised OPLS-DA modeling. The PCA score plot (Figure 6A) demonstrated distinct serum metabolic profiles among the three groups, with the HGyp group clustering toward the control group. OPLS-DA further enhanced separation resolution, revealing clearly segregated clusters for the control, APAP, and HGyp groups in independent regions of the score plots (Figure S5A,B). We subsequently employed OPLS-DA to specifically compare metabolic profile differences between the APAP vs. control group and HGyp vs. APAP group (Figure S5C,D). The reliability of OPLS-DA models was confirmed by permutation testing, where left-side R2 and Q2 values were lower than the initial right-side values, indicating minimal overfitting risk. Figure S5E,F visualize the global distribution of differential metabolites, with each point representing an ion. Ions distant from the origin were identified as potential biomarkers, demonstrating distinct expression trends between comparative groups.

#### 3.5.2. Identification of Potential Biomarkers in Serum

**Figure 5:**
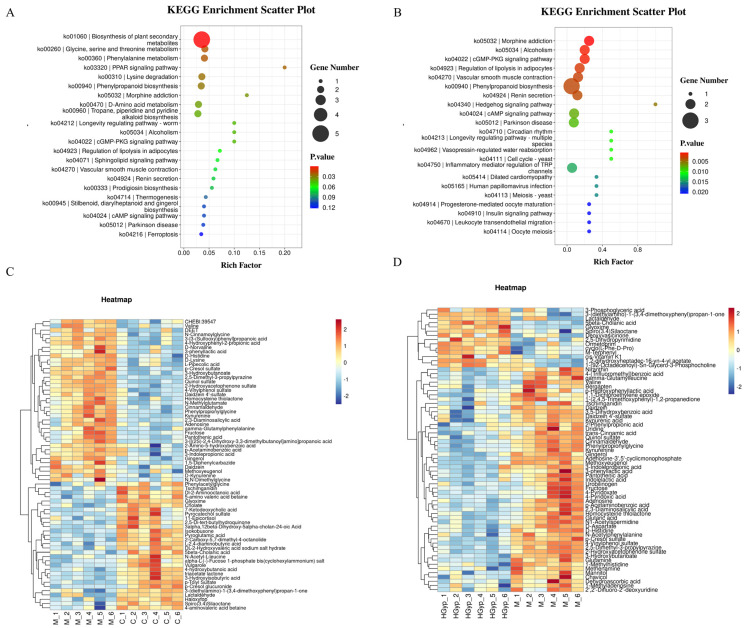
Main metabolic pathways of differential metabolites in serum of C vs. M (**A**). and HGyp vs. M (**B**). Heat maps of differential metabolites in serum of C vs. M (**C**) and HGyp vs. M (**D**). (Control group: C; APAP group: M; HGyp-H group: HGyp.)

**Figure 6:**
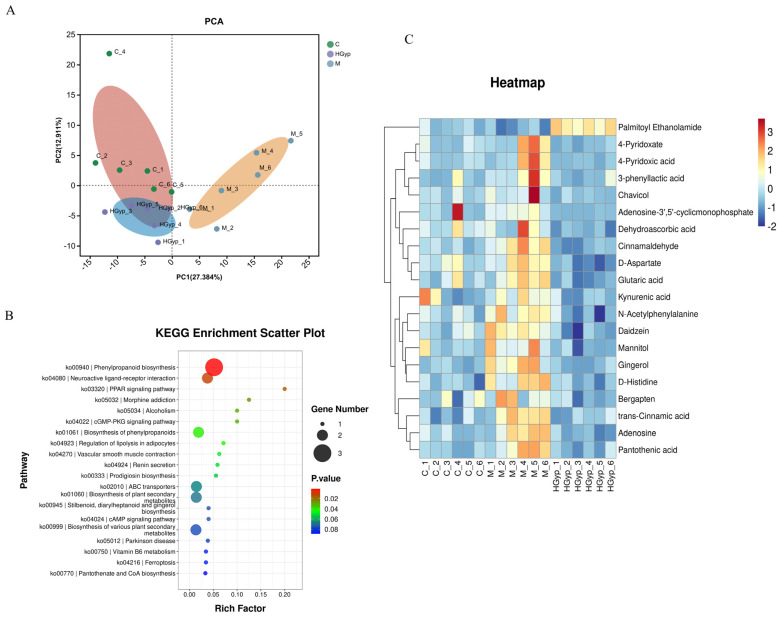
PCA score plots of the control, model and HGyp groups in serum samples (**A**). Main metabolic pathways of differential metabolites in serum (**B**). Heat maps of differential metabolites in serum (**C**). (Control group: C; APAP group: M; HGyp-H group: HGyp.)

To identify potential biomarkers, we analyzed characteristic alterations of endogenous metabolites among the control, APAP, and HGyp groups. Differentially expressed metabolites were identified based on VIP > 1.0, FC ≥ 1.50 or ≤ 0.75, and *p* < 0.05. For water-soluble and neutral metabolites, comparative analysis revealed 21 potential biomarkers in the APAP group vs. controls, including 7 upregulated and 14 downregulated (Table S3). For all the metabolites, comparative analysis revealed 25 potential biomarkers in the APAP group vs. controls, including 9 upregulated and 16 downregulated (Table S4). The relative abundance patterns of these candidate biomarkers were visualized in heatmaps (Figure 5C,D and Figure 6C). Notably, HGyp treatment restored the levels of all 25 dysregulated metabolites toward normal ranges, suggesting these metabolites may serve as potential biomarkers for evaluating the hepatoprotective effects of HGyp.

#### 3.5.3. Metabolic Pathway Analysis

The identified serum potential biomarkers were imported into MetaboAnalyst 5.0 for pathway analysis, revealing 48 significantly altered metabolic pathways in serum (Figure 5A,B and Figure 6B): phenylpropanoid biosynthesis, neuroactive ligand–receptor interaction, the PPAR signaling pathway, morphine addiction, the cGMP-PKG signaling pathway, the regulation of lipolysis in adipocytes, ferroptosis, the inflammatory mediator regulation of TRP channels, D-amino acid metabolism, and tryptophan metabolism as potentially key pathways mediating HGyp’s therapeutic effects against liver injury.

### 3.6. Network Pharmacology Analysis

#### 3.6.1. Target Prediction and the Intersection Between HGyp and LI

**Figure 7:**
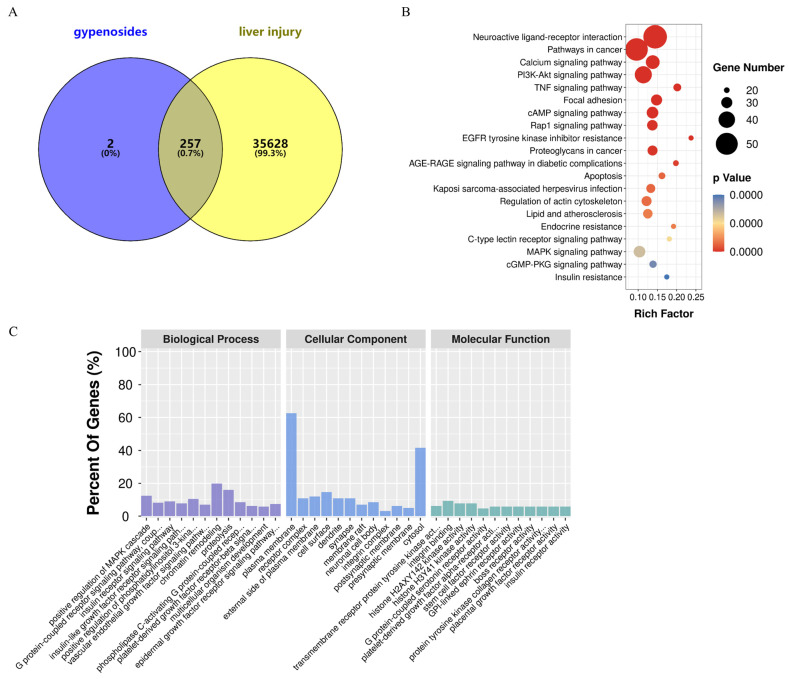
A network pharmacology analysis of HGyp against LI. (**A**) A Venn diagram of the intersection of the targets between HGyp and LI. (**B**) A KEGG pathway enrichment analysis of the top 20 pathways is listed based on the *p* values. (**C**) A GO enrichment analysis of the top 12 terms of the biological process, cellular component, and molecular function is displayed.

HGyp was found to contain nine identified chemical constituents. A total of 38 compounds were detected in the in vivo migration study of heat-processed *G. pentaphyllum* ethanol extract, including 9 prototype compounds that entered the bloodstream unchanged: gypenoside LVI, gypenoside XLVI, damulin A, damulin B, gypenoside L, gypenoside LI, 20(S)-ginsenoside Rg3, 20(R)-ginsenoside Rg3, and ginsenoside Rd. Through comprehensive target screening, we collected 259 component-related targets and 35,885 liver injury-associated targets. Venn diagram analysis (Figure 7) revealed 257 intersecting targets that are directly involved in the therapeutic effects of HGyp against liver injury.

#### 3.6.2. GO and KEGG Enrichment Analysis

A DAVID-based analysis of 257 targets (Figure 7A) revealed extensive functional associations, identifying 1375 biological processes, 120 cellular components, and 224 molecular functions (*p* < 0.05). The top 12 enriched GO terms are shown in Figure 7C. KEGG pathway mapping demonstrated 155 significant entries (*p* < 0.05), with PI3K-Akt, TNF, Rap1, MAPK, and apoptotic signaling emerging as the core pathways mediating HGyp’s hepatoprotective effects. The top 20 most relevant pathways are displayed in Figure 7B.

#### 3.6.3. Potential Effective Components and Core Targets Screening

**Figure 8:**
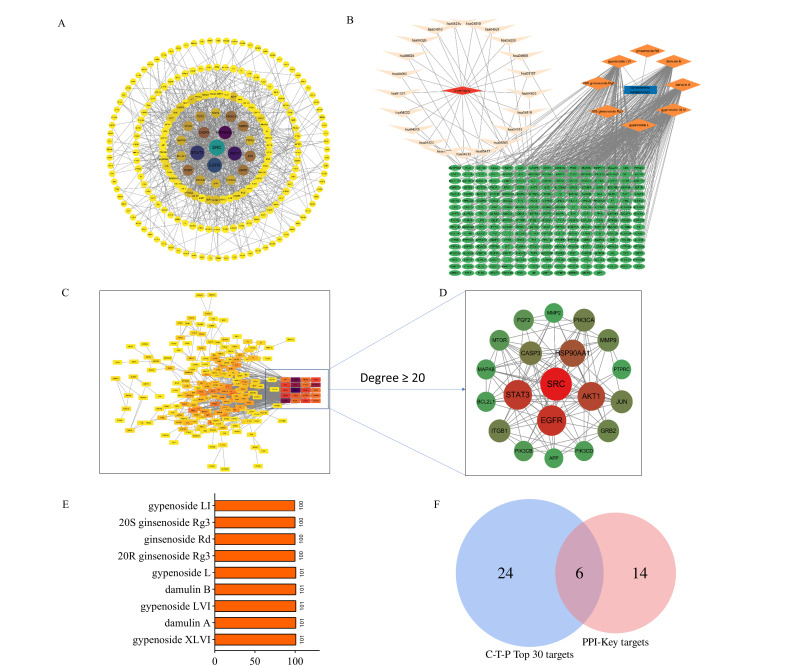
Network pharmacology analysis of HGyp against LI. (**A**) PPI network. (**B**) Network diagram of C-T-P. (**C**,**D**) Key targets in PPI network. (**E**) Potential effective components of HGyp and degree values. (**F**) Venn diagram of targets in PPI and C-T-P.

Through integrated protein–protein interaction and compound–target–pathway (C-T-P) network analyses, we identified the key bioactive constituents and their primary therapeutic targets. The PPI network (Figure 8A) was filtered by topological parameters to identify 20 highly interconnected targets (Figure 8C,D). Furthermore, we integrated key KEGG pathways, corresponding targets of HGyp, and migrated components to establish a comprehensive C-T-P network through Cytoscape (Figure 8B). Degree centrality analysis revealed that gypenoside L/LI, gypenoside LVI/XLVI, damulin A/B, 20(S)-ginsenoside Rg3, 20(R)-ginsenoside Rg3, and ginsenoside Rd exhibited a degree > 100, identifying them as potential active components for the HGyp treatment of LI (Figure 8E). Intersection analysis (Figure 8F) demonstrated an overlap between the 20 high-connectivity targets from PPI and the top 30 degree-ranked targets from the C-T-P network, ultimately identifying six core targets: GRB2, MMP2, FGF2, STAT3, CASP3, and HSP90AA1.

#### 3.6.4. Effective Component Screening and Molecular Docking Validation

**Figure 9:**
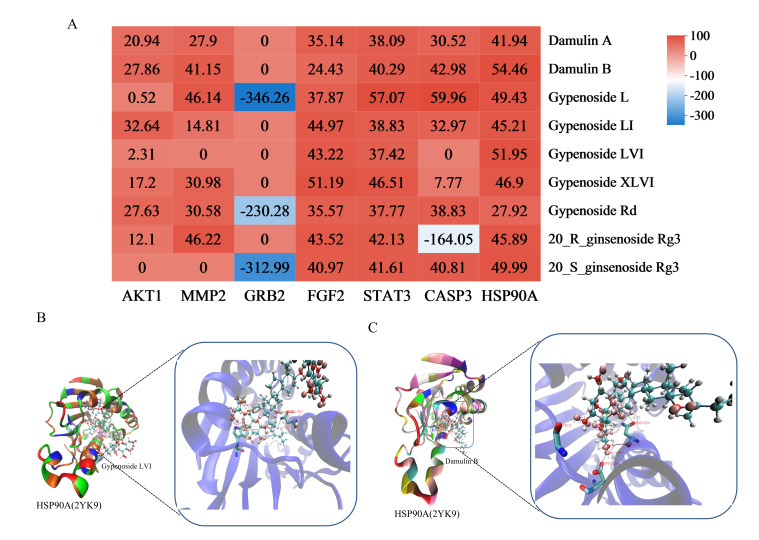
Heat maps of molecular docking results (**A**). The bluer the color, the smaller the corresponding value, indicating greater docking strength. The co-crystal structure of gypenoside LVI and HSP90A (**B**). Dumulin B and HSP90A (**C**).

Molecular docking was performed between the six core target proteins (receptors) identified through network pharmacology and the high-degree compounds (ligands). The binding affinity results are presented in Figure 9A; deeper red coloration indicates more stable connections with higher binding affinity. Among the nine components evaluated with varying docking scores, those exhibiting strong binding affinity with core target proteins were considered potential active constituents. Integrated analysis with the network pharmacology results identified the top five most promising compounds: gypenoside L, damulin B/A, and gypenoside LVI/XLVI. Structural analysis revealed the formation of hydrogen bonds and Van der Waals forces between these active compounds and key residue sites in the core targets (Figure 9B,C, Table S5). These results demonstrate that gypenoside L/LI, damulin B/A, and gypenoside LVI/XLVI exhibit excellent binding capacity with core LI targets (MMP2, FGF2, STAT3, CASP3, and HSP90AA1), suggesting their potential as the primary bioactive components responsible for HGyp’s therapeutic effects.

### 3.7. Integration of Metabolomics and Network Pharmacology Analysis

**Figure 10:**
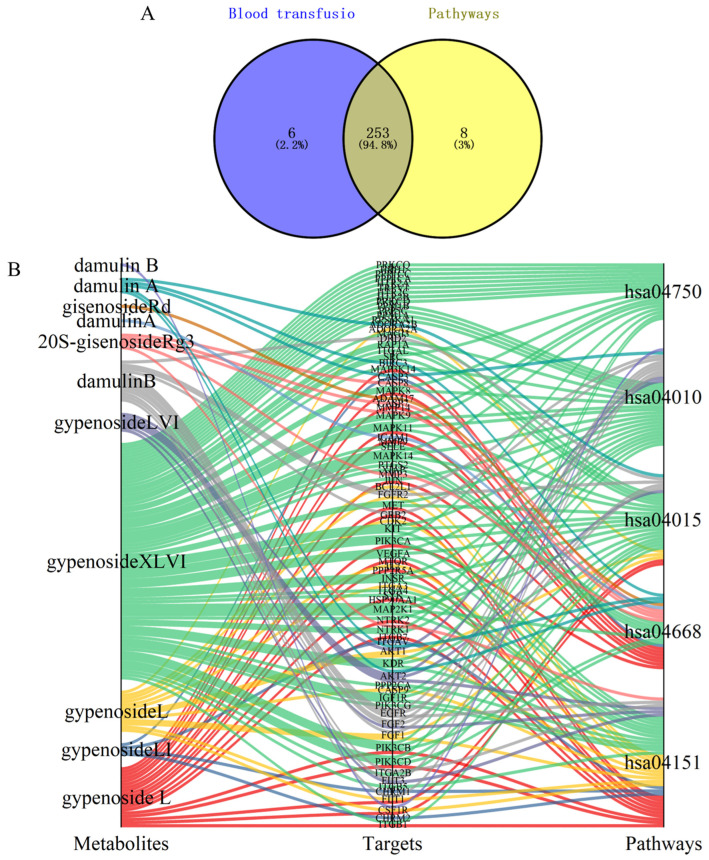
(**A**) Venn diagram of overlapping targets between metabolites and five pathways related to LI. (**B**) Network diagram of metabolite–target–pathway.

**Figure 11:**
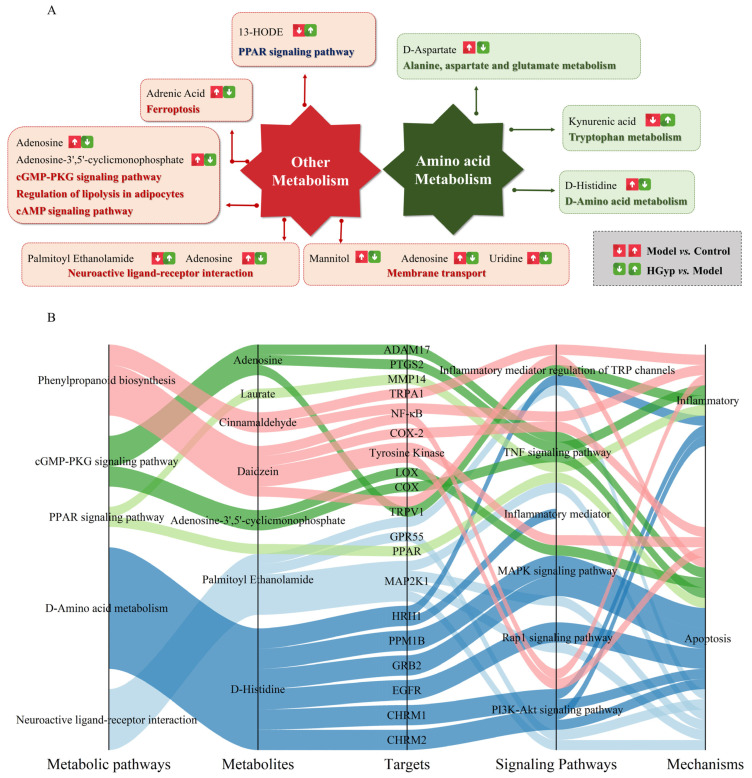
(**A**) The construction of the altered metabolic pathway network of HGyp-treated LI mice based on the KEGG pathway database. (**B**) The relationship between metabolic pathways regulated by HGyp, metabolites regulated by HGyp, the validated targets from network pharmacological prediction, pathways associated with LI, and the related mechanisms of HGyp against LI.

Through Swiss Target Prediction database analysis, we identified 259 potential targets for the nine prototype blood-absorbed components. Furthermore, five key LI-associated pathways were predicted: PI3K-Akt (hsa04151), TNF (hsa04668), Rap1 (hsa04015), MAPK (hsa04010), and the inflammatory mediator regulation of TRP channels (hsa04750). Intersection analysis between pathway targets and component targets yielded 253 overlapping targets (Figure 10A). A multi-layered interaction network was established, connecting 9 key metabolites with 79 consensus targets across 5 central signaling pathways (Figure 10B), revealing the integrated pharmacological framework of HGyp’s therapeutic effects. Additionally, we developed an integrated network connecting HGyp-affected metabolites, metabolic pathways, core targets, LI-related signaling pathways, and their mechanistic associations (Figure 11), providing a systems-level understanding of HGyp’s therapeutic actions.

### 3.8. Effects of HGyp on Liver-Related Protein Expression

**Figure 12:**
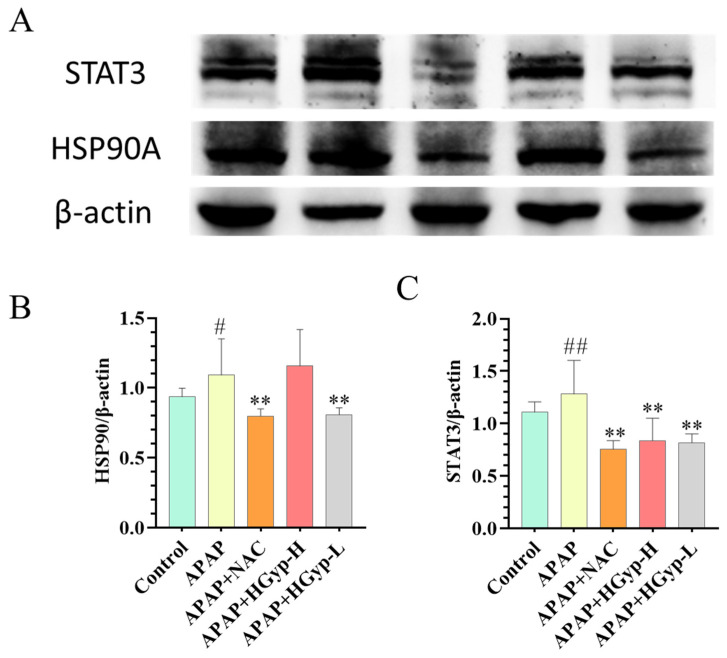
The influence of HGyp on the expression of HSP90A and STAT3 in CI mice. (**A**) Western blot images. (**B**) HSP90 expression level. (**C**) STAT3 expression level. Data are shown as mean ± SD for *n* = 3. ^#^ *p* < 0.05, ^##^ *p* < 0.01 vs. ND group. * *p* < 0.05, ** *p* < 0.01 vs. HFD group. Original images can be found in Supplementary Materials.

To investigate the mechanism of HGyp against liver injury, we examined hepatic HSP90 and STAT3 expression by WB. The APAP group significantly suppressed both HSP90 (*p* < 0.01) and STAT3 protein levels in liver tissues in comparison to the control group. In contrast, HGyp administration markedly upregulated the expression of HSP90 (*p* < 0.01) and STAT3 (*p* < 0.01) compared to the model group (Figure 12 and S6). These results demonstrate that HGyp effectively reverses the APAP-induced suppression of hepatoprotective proteins.

## 4. Discussion

*G. pentaphyllum*, a traditional Chinese medicine and ethnomedicine, has been clinically used for years to treat hyperlipidemia, fatty liver, and hepatitis. Our previous study demonstrated that heat processing increased the content and diversity of saponins in *G. pentaphyllum*. However, the material basis and underlying mechanisms of heat-processed *G. pentaphyllum* in hepatoprotection remain unclear. Therefore, we employed serum pharmacochemistry, metabolomics, and network pharmacology to identify the active components of HGyp and their molecular mechanisms against LI. In this study, HGyp significantly alleviated APAP-induced liver damage in mice, as evidenced by improvements in the liver index, serum biochemical markers, and histopathology. Serum pharmacochemical analysis detected 38 absorbed components in vivo, including 16 saponin prototypes and 11 metabolites. Network pharmacology predicted the targets of nine prototype blood-absorbed components related to LI. Integrated PPI analysis and C-T-P network screening identified nine potential active compounds and five key signaling pathways. Furthermore, molecular docking and Western blotting validated the strong binding affinity between these active components and core targets (GRB2, FGF2, MMP2, STAT3, CASP3, and HSP90A), demonstrating the efficacy of this multi-omics approach in target prediction and compound screening. This study not only provides experimental evidence for ethnomedicine research but also holds potential clinical significance. However, this study did not directly measure the concentrations of HGyp-derived constituents in liver tissue. Therefore, it remains uncertain to what extent each identified prototype compound or metabolite accumulates in the liver or undergoes rapid hepatic metabolism. It is possible that the observed hepatoprotective effects are driven by certain components with favorable liver distribution characteristics or by active metabolites formed within the liver. Future studies employing tissue pharmacokinetics (PK) and imaging techniques such as mass spectrometry imaging (MSI) will help quantify the spatial and temporal distribution of these saponins in the liver. Such data will further strengthen the association between specific compounds and the observed pharmacological effects. Notably, the model group exhibited increased body weight, likely due to pathological fluid accumulation from severe hepatic edema and ascites masking actual tissue loss, alongside a significantly elevated liver index. Histopathological findings confirmed hepatocellular injury, interstitial edema, and sinusoidal congestion. Treatment significantly mitigated both body weight gain and the liver index, suggesting an amelioration of hepatic edema and injury.

Gene regulation and metabolic processes represent the ultimate outcomes of cellular activity [37]. In this study, UPLC-MS-based untargeted metabolomics was employed to investigate the potential therapeutic mechanisms of HGyp in LI. HGyp treatment effectively reversed APAP-induced alterations in serum metabolites in mice. A total of 21 biomarkers were identified in the serum.

Metabolomic profiling identified significant disturbances in several key metabolic pathways, particularly in fatty acid oxidation and aromatic compound metabolism, which were intricately linked to the observed hepatic injury (Figure 11A). Metabolites such as 13-HODE and 9-HODE, as prominent products of linoleic acid peroxidation, were markedly elevated. These hydroxyoctadecadienoic acids are not only robust markers of oxidative stress but also act as pro-inflammatory lipid mediators, capable of activating neutrophils and exacerbating hepatocyte damage [38,39]. 13-HODE, a product of linoleic acid peroxidation, is known to exacerbate oxidative stress and inflammation by inhibiting catalase and activating NF-κB and MAPK pathways. The normalization of these metabolites by HGyp treatment underscores its role in counteracting both oxidative and inflammatory components of APAP hepatotoxicity, further supporting its multi-faceted hepatoprotective profile. It is important to note that our untargeted metabolomics approach, while effective for capturing hydrophilic and moderately lipophilic metabolites, does not provide a comprehensive profile of the entire lipidome. Similarly, the increase in adrenic acid further signifies enhanced lipid peroxidation, a well-established mechanism in the progression of non-alcoholic steatohepatitis and DILI [40]. Concurrently, the tryptophan pathway metabolite kynurenic acid was significantly upregulated. As a driver of the inflammatory response, its accumulation is closely associated with the activation of immune cells and the aggravation of hepatic inflammation and fibrosis [41]. The disruption in fatty acid metabolism was also evidenced by the accumulation of medium-chain fatty acids like laurate/dodecanoic acid, which can impose metabolic strain on hepatocytes, and the perturbation of the omega-3/omega-6 balance reflected by changes in docosahexaenoic acid and Cis-8,11,14-Eicosatrienoic acid. Furthermore, alterations in plant-derived xenobiotics were noted. trans-Cinnamic acid and its derivative cinnamaldehyde, while potentially antioxidative at low levels, have been implicated in idiosyncratic liver toxicity at high doses or upon prolonged exposure, likely contributing to the initial cellular stress [42]. The endogenous metabolites palmitoyl ethanolamide and adenosine exhibited compensatory elevations, which are interpreted as part of the body’s counter-regulatory response to injury, given their well-documented anti-inflammatory and cytoprotective roles in the liver [43]. In summary, this metabolite panel paints a coherent picture of hepatic injury characterized by pervasive oxidative stress, inflammatory signaling, and compromised cellular homeostasis.

The metabolomics profile revealed significant alterations in polar lipid species, particularly lysophospholipids (e.g., LysoPCs) and fatty acid metabolites, which were closely associated with the therapeutic effects of HGyp on LI. These changes point to disruptions in related pathways such as glycerophospholipid remodeling and fatty acid metabolism (Table S4). Lysophosphatidylcholines such as LysoPC (18:3), LPC 18:3, LPC 22:5, and PC (18:2/0:0) exhibited significant changes in DILI. As products of the phospholipase A2-mediated hydrolysis of phosphatidylcholines, the elevated levels of these LysoPCs reflect enhanced phospholipid remodeling and membrane damage, which are known to contribute to hepatocyte membrane damage and inflammatory responses [44]. Sphingosine-1-phosphate, which regulates hepatocyte apoptosis or fibrosis, was markedly elevated in DILI [45]. The gut microbiota-derived metabolite trans-cinnamic acid may influence DILI progression by modulating inflammatory responses [46]. Pantothenic acid served as an indirect biomarker of energy metabolism disruption [47]. From the analysis of the entire metabolic products, the changes in lipid levels may also be part of the mechanism of action of HGyp.

Thus, APAP-induced liver injury involves hepatocyte apoptosis, lipid metabolism, and inflammatory responses, and HGyp demonstrates a regulatory role in reversing these pathological processes.

As the primary site of amino acid catabolism, the liver critically maintains systemic amino acid balance through enzymatic conversions such as phenylalanine hydroxylase-mediated tyrosine synthesis [48]. The hepatic metabolism of phenylalanine/tyrosine directly reflects liver function [49], as evidenced by their strong correlation with transaminase (AST/ALT) activity profiles in our study. Figure 11A and Tables S4 and S5 demonstrate a marked accumulation of phenylalanine and D-amino acid metabolic intermediates in APAP-intoxicated mice, reflecting hepatic metabolic dysfunction, suggesting that phenylalanine-related metabolic disturbances may impair normal liver function. In contrast, the HGyp-treated group exhibited reduced levels of these metabolites compared to the APAP group. HGyp appears to exert hepatoprotective effects against APAP toxicity through the regulation of amino acid metabolic pathways.

A complex and bidirectional regulatory relationship exists between metabolites and genes, wherein genes influence metabolites and vice versa. In this study, we employed an integrated approach combining network pharmacology and metabolomics to establish connections between differential metabolites and gene targets. Figure 11B illustrates potential associations among HGyp-affected metabolites, metabolic pathways, altered compound targets in vivo, LI-related signaling pathways, and their underlying mechanisms. Caspase-3 (CASP3), a crucial mediator of pro-apoptotic activity, is intimately associated with morphological and biochemical changes during apoptosis and is considered central to the apoptotic system [50]. HGyp administration reversed APAP-induced sphingomyelin alterations, potentially preventing apoptosis and attenuating hepatic fibrosis progression. Collectively, these findings suggest plausible associations between HGyp-regulated targets and metabolites in the therapeutic intervention.

To elucidate the molecular mechanism of HGyp in LI treatment, we identified several key targets (AKT1, MMP2, GRB2, FGF2, STAT3, CASP3, HSP90AA1, and TNF) from LI-related signaling pathways. This study revealed that HGyp modulates diverse biological processes including anti-inflammatory effects, cell proliferation, and apoptosis. These findings suggest that HGyp exerts its multi-target and multi-pathway hepatoprotective effects through the regulation of the PI3K-Akt, MAPK, Rap1, TRP, and TNF signaling pathways.

Tissue injury triggers the release of pro-inflammatory cytokines, initiating a cascade of inflammatory responses primarily mediated by hepatic macrophages and Kupffer cells [51]. APAP stimulation has been reported to activate the NF-κB inflammatory pathway, thereby promoting the expression of pro-inflammatory factors [52]. The MAPK cascade, particularly the p44/42 signaling axis, orchestrates fundamental cellular processes ranging from proliferation to apoptosis in response to diverse stimuli (e.g., thermal/osmotic stress). Concurrently, HSP90 functions as a master regulatory chaperone, ensuring proteostasis through protein folding, stabilization, and targeted degradation while modulating critical growth-related signaling networks [53]. In the present study, APAP-treated mice exhibited elevated hepatic HSP90 levels, whereas HGyp treatment effectively reduced HSP90 protein expression, subsequently inhibiting apoptosis and mitigating liver injury.

In this study, acute liver injury induced hepatocyte apoptosis and elevated the expression of pro-inflammatory cytokines, indicating the activation of the innate immune system. Members of the interleukin (IL) family (IL-6, IL-13, and IL-22) effectively activated STAT3 during hepatic repair. STAT3 serves as a crucial signaling molecule that directly or indirectly regulates the expression of key genes involved in liver regeneration [54]. STAT3 primarily interacts with JAK tyrosine kinases and participates in the downstream signal transduction of extracellular signals, exhibiting potent anti-apoptotic and mitogenic effects [55]. Pro-inflammatory cytokines trigger the JAK2/STAT3 signaling cascade through sequential phosphorylation events, ultimately modulating inflammatory gene expression profiles [56]. Our results demonstrated that HGyp significantly suppressed APAP-induced STAT3 expression, effectively preventing inflammatory progression. While APAP intoxication triggered TNF-mediated inflammatory signaling, HGyp administration significantly suppressed TNF-α production, demonstrating potent anti-inflammatory activity.

The nine compounds gypenoside L/LI, gypenoside LVI/XLVI, damulin A/B, 20(S)-ginsenoside Rg3, 20(R)-ginsenoside Rg3, and ginsenoside Rd exhibited strong binding affinity to the core targets of LI. Following network analysis and reverse screening via molecular docking, they were identified as potential active constituents of HGyp. Notably, six of these compounds, gypenoside L/LI, gypenoside LVI/XLVI, and damulin A/B, demonstrated particularly promising interactions. Previous studies have revealed that damulin B exerts anti-apoptotic and antioxidant effects by modulating AMPKα1 and ROS [57]. Meanwhile, gypenoside L and gypenoside LI were found to upregulate COX2 expression while downregulating cPLA2 and CYP1A1, thereby reducing arachidonic acid levels and inducing apoptosis. Additionally, gypenoside L and LI increased DUSP1, p-JUN, and p-JNK while decreasing p-MEK1/2, p-ERK, and p-P38 [58].

The multi-targeted mechanisms of HGyp, particularly its efficacy in mitigating the secondary inflammatory and metabolic phases of APAP injury, suggest its potential as a complementary or alternative therapeutic strategy to the current standard of care NAC. A critical limitation of NAC is the narrow therapeutic window; its role as an ROS scavenger and GSH precursor is most effective during the initial oxidative burst, with diminishing returns upon the establishment of mitochondrial failure and necrosis in late-stage injury. Furthermore, the translational potential of NAC for chronic liver diseases is hampered by suboptimal pharmacokinetics, specifically low bioavailability and rapid clearance, thus preventing the sustained therapeutic concentrations needed and further limiting its use in conditions such as MASLD and hepatic fibrosis [59]. Our results, however, demonstrate that HGyp offers a multi-targeted mechanism, simultaneously tackling inflammation (via the downregulation of TNF-α/STAT3) and metabolic dysregulation (via the restoration of glycerophospholipid and sphingolipid metabolism). This key difference implies that HGyp could complement NAC by acting on later stages of injury, potentially prolonging the treatment window beyond the point of GSH depletion. Notably, the variety of bioactive saponins in HGyp may also provide superior pharmacokinetics, allowing for continuous multi-pathway modulation. Therefore, HGyp represents a promising alternative for subacute or chronic liver diseases like MASLD and fibrosis, scenarios where NAC’s efficacy is often constrained.

Based on the above findings, the protective effects of HGyp against LI are primarily associated with lipid homeostasis regulation, anti-inflammatory activity, and the modulation of apoptosis. The therapeutic mechanism of HGyp in LI fully reflects its multi-component, multi-target, and multi-pathway characteristics. However, this study has certain limitations. Databases, as a major component of network pharmacology research, inherently contain biases, necessitating frequent updates to maintain data accuracy. Further investigation is required to validate the mRNA expression and protein levels of the core targets identified through network pharmacology. Additionally, the target interactions and molecular regulatory networks need to be experimentally verified both in vitro and in vivo. Although the screened active components help elucidate the mechanisms underlying LI treatment, their biological activity remains insufficiently studied. One limitation of this study is the absence of a direct comparison between heat-treated HGyp and untreated *G*. *pentaphyllum* extract in the acetaminophen-induced injury model. Therefore, although we have clearly identified the active constituents and mechanism of action of the heat-treated form, the present data do not allow us to quantitatively conclude that HGyp is superior to the untreated form in this specific application. However, the primary objective of this study was not to compare efficacy but to systematically elucidate the material basis and mechanism of action of heat-treated *G. pentaphyllum*. Our team’s previous chemical analyses [24,28] clearly demonstrated that heat treatment significantly alters the composition of saponins, generating unique compounds and increasing the content of specific gypenosides (such as L, LI, LVI, and XLVI), which are identified herein as the core bioactive components. Thus, the observed hepatoprotective effects are intrinsically linked to this unique chemical profile resulting from heat treatment. Future studies directly comparing the pharmacological profiles are warranted and will constitute an important direction for our subsequent research. This study is missing of a direct comparison between HGyp and untreated *G. pentaphyllum* extract in the APAP model. Therefore, although we have clearly identified the active constituents and mechanism of action of HGyp, the present data do not allow us to quantitatively conclude that HGyp is superior to the untreated form in this specific application. The primary objective of this study was systematically elucidate the material basis and mechanism of action of HGyp. Our team’s previous chemical analyses [19,24,28,29,30,31,32,33] clearly demonstrated that heat treatment significantly alters the composition of saponins, generating unique compounds and increasing the content of specific gypenosides (such as L, LI, LVI, and XLVI), which are identified herein as the core bioactive components. Thus, the observed hepatoprotective effects are intrinsically linked to this unique chemical profile resulting from heat treatment. Future studies directly comparing the pharmacological profiles are warranted and will constitute an important direction for our subsequent research. Therefore, our subsequent research will focus on addressing these limitations.

## 5. Conclusions

In this study, we employed an integrated approach combining serum pharmacology, network pharmacology, and metabolomics to investigate the active components and mechanisms of action of traditional ethnic medicines. Our findings demonstrate that HGyp effectively alleviates APAP-induced LI. Nine active components (gypenoside L/LI, gypenoside LVI/XLVI, damulin A/B, 20(S)-ginsenoside Rg3, 20(R)-ginsenoside Rg3, and ginsenoside Rd), six key targets (GRB2, FGF2, MMP2, STAT3, CASP3, and HSP90A), five signaling pathways (PI3K-Akt, MAPK, Rap1, TRP, and TNF signaling pathways), and 12 related metabolic pathways were identified as potentially playing critical roles in the therapeutic mechanism of HGyp. The protective effects of HGyp against LI may involve lipid metabolism and anti-inflammatory and anti-apoptotic mechanisms. Immunoblotting verified the presence of two key target proteins. Further in vitro and in vivo studies, along with more detailed compositional analyses, are required to validate these targets, their associated mechanisms, and the active constituents. This research strategy provides a novel approach for modernizing the study of traditional ethnic medicines.

## Figures and Tables

**Table 1:** The effects of HGyp on the body weight, heart, liver, spleen, lung and kidney weights of mice with liver injury induced by APAP (*n* = 10).

Group	Control	APAP	APAP + NAC	APAP + HGyp-H	APAP + HGyp-L
Body weight (g)	22.450 ± 0.433	24.220 ± 0.491 ^#^	24.707 ± 0.443	23.037 ± 0.473	23.637 ± 0.477
Heart (g)	0.147 ± 0.084	0.141 ± 0.002	0.156 ± 0.009	0.142 ± 0.009	0.149 ± 0.004
Liver (g)	0.939 ± 0.026	1.246 ± 0.033 ^##^	1.216 ± 0.154	1.042 ± 0.032 ^**^	1.105 ± 0.035 ^**^
Spleen (g)	0.073 ± 0.002	0.095 ± 0.003 ^##^	0.082 ± 0.001 ^**^	0.078 ± 0.003 ^**^	0.082 ± 0.001 ^**^
Lung (g)	0.144 ± 0.004	0.154 ± 0.007	0.155 ± 0.005	0.158 ± 0.007	0.144 ± 0.004
Kidney (g)	0.367 ± 0.009	0.391 ± 0.015	0.400 ± 0.010	0.376 ± 0.014	0.389 ± 0.008

Data are shown as mean ± SD for *n *= 10. ^#^ *p* < 0.05, ^##^ *p* < 0.01 vs. Control group. * *p* < 0.05, ** *p* < 0.01 vs. APAP group.

## Data Availability

The original contributions presented in this study are included in the article/Supplementary Materials. Further inquiries can be directed to the corresponding author.

## Supplementary Materials

The following supporting information can be downloaded at https://www.mdpi.com/article/10.3390/biom15111555/s1, Figure S1: Gypenoside LVI full-waveform detection; Figure S2: Calibration curve of gypenoside LVI; Figure S3: Mass spectrum of peak at retention time of HGyp; Figure S4: Thermal treatment of the ethanol extract of *Gynostemma pentaphyllum* and its blood component TIC diagram; Figure S5: The influence of HGyp on the metabolites in mouse serum; Figure S6: Effect of HGyp on the STAT3 and HSP90; original uncropped blots; Table S1: Gypenosides identified by LC-MS; Table S2: The differential metabolites in the serum of rats; Table S3: Targeted analysis of water-soluble and medium-polarity metabolites using UHPLC-QE MS identified key differential metabolites; Table S4: Analysis of the entire metabolome detected by UHPLC-QE MS revealed key differential metabolites; Table S5: Binding energy of gypenosides based on the Gold docking simulation (kcal/mol); File S1: Original Western blot images.

## Abbreviations

The following abbreviations are used in this manuscript:

TCM	traditional Chinese medicine
HGyp	heat-processed gypenosides
AST	aminotransferase
ALT	alanine aminotransferase
SOD	superoxide dismutase
TNF-α	tumor necrosis factor-α
LI	liver injury
NAC	N-acetylcysteine
APAP	acetaminophen
GOLD	Genetic Optimisation for Ligand Docking
13-HODE	13-hydroxyoctadecadienoic acid
