# Supplementary material for: Multi-Omics Reveals Active Components and Mechanisms of Heat-Processed Gypenosides Hepatoprotective Against APAP Injury
Source: Biomolecules. 2025 Nov 5;15(11):1555. doi: 10.3390/biom15111555 (PMC12650016; doi:10.3390/biom15111555)
Supplement: Supplementary file 1 [file biomolecules-15-01555-s001.zip › biomolecules-3966887-supplementary.pdf]

Multi-Omics Reveals Active Components and Mechanisms of Heat-Processed Gypenosides Hepatoprotective Against APAP Injury

Peng Xie [1,*], Qiu-Ru Li [1], Shu Jiang [1], Miao Sun [1], Yu-Duan [3], Chang-Ping Hu [1] and Xiang-Lan Piao [2,*]

[1] Shanxi Provincial Department-Municipal Key Laboratory Cultivation Base for Quality Enhancement and Utilization of Shangdang Chinese Medicinal Materials, School of Pharmacy, Changzhi Medical College, Changzhi, Shanxi, 046000, PR, China
[2] School of Pharmacy, Minzu University of China, Beijing, 100081, China
[3] China Academy of Traditional Chinese Medicine, Beijing 100081, China
* Correspondence: pxie2024@czmc.edu.cn

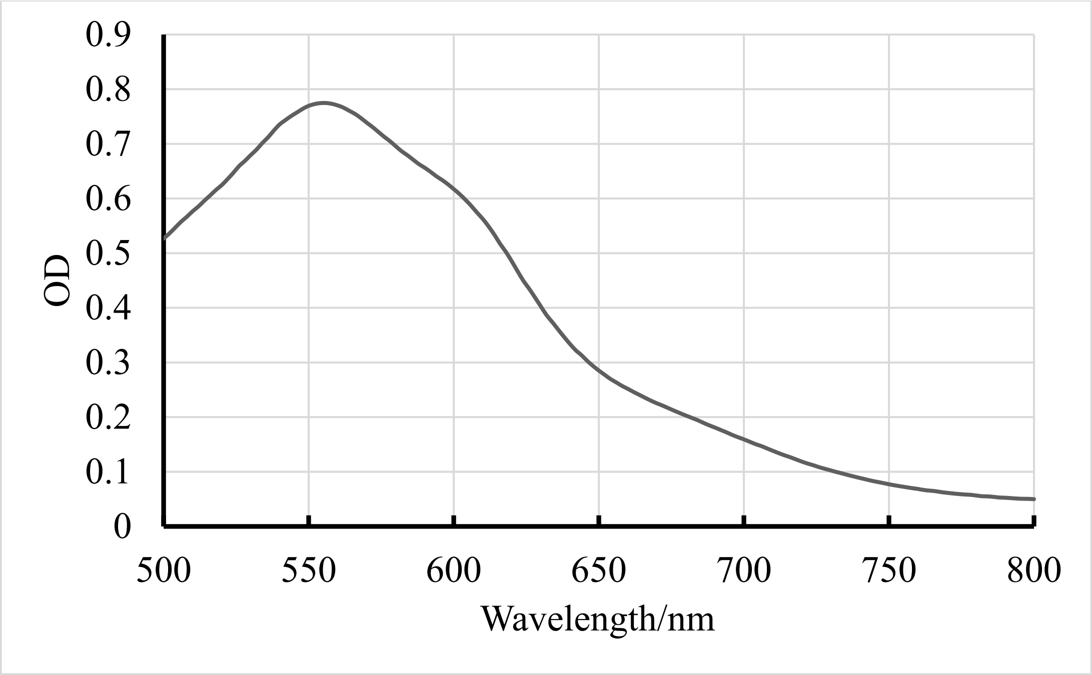

Figure S1. Gypenoside LVI full-waveform detection.

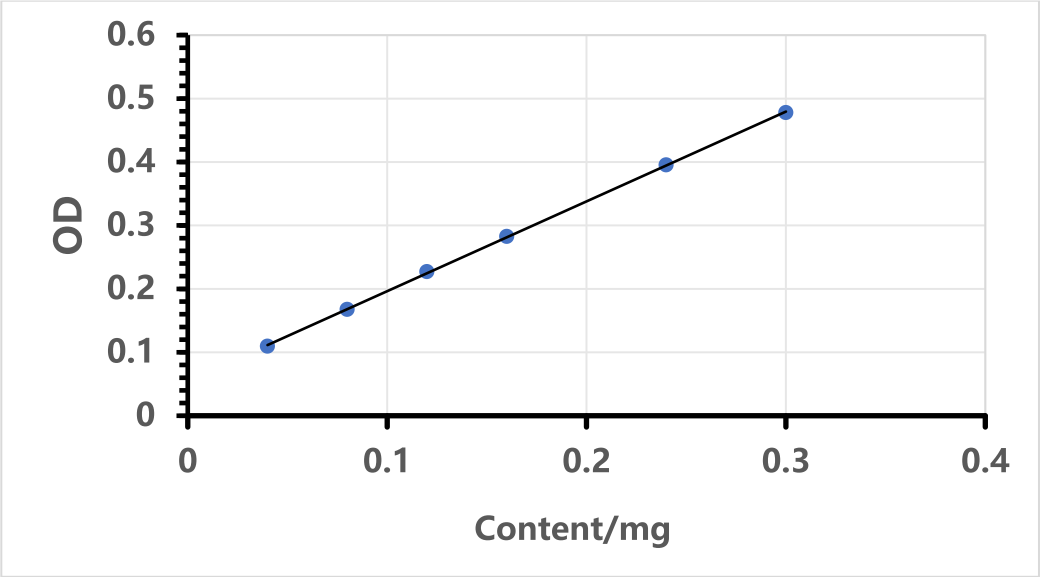

Figure S2. Calibration curve of gypenoside LVI.

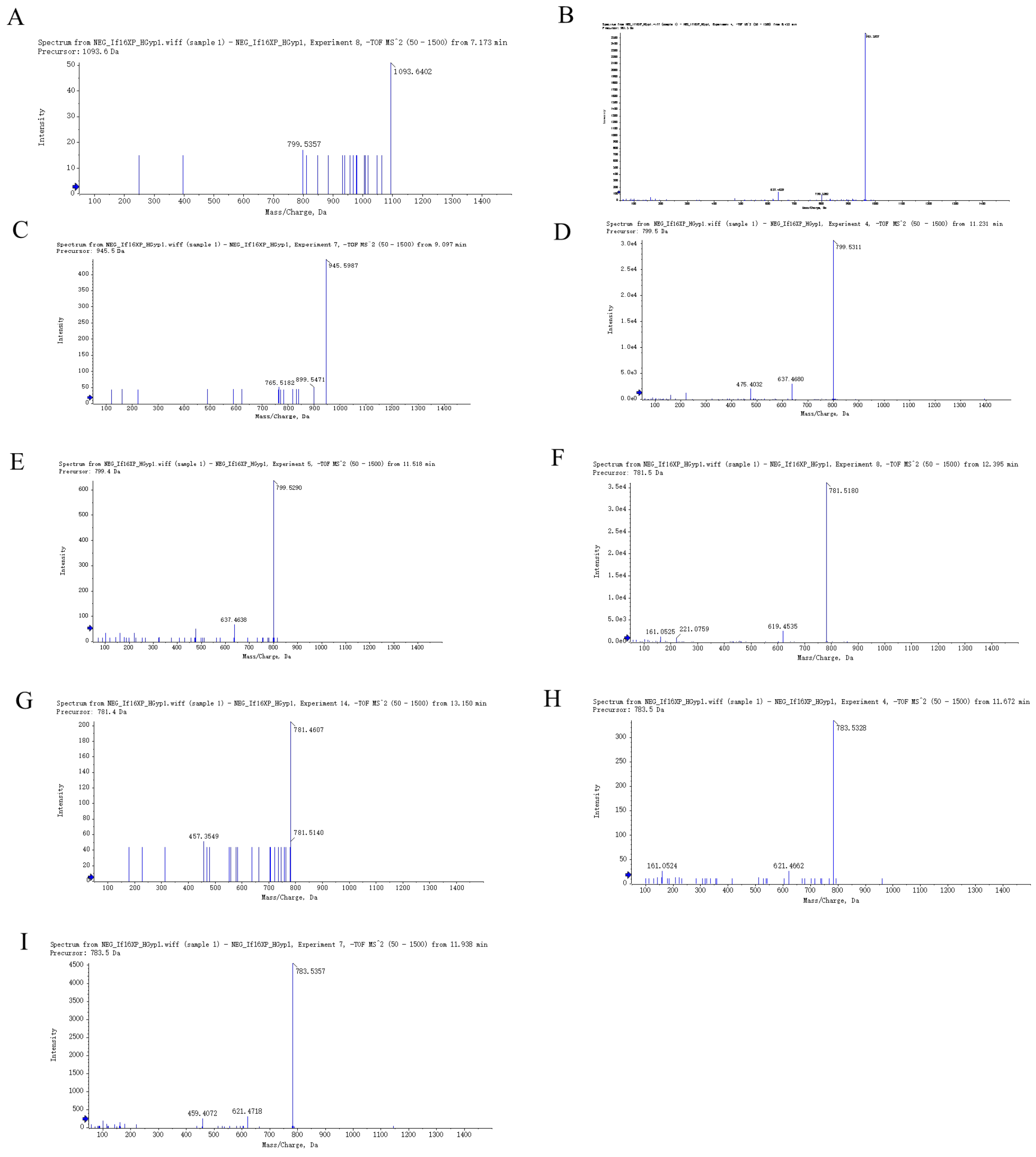

Figure S3. Mass spectrum of peak at retention time of HGyp. (A) gypenoside LVI, (B) gypenoside XLVI, (C) ginsenoside Rd, (D) gypenoside L, (E) gypenoside LI, (F) damulin B, (G) damulin A, (H) 20(S)-ginsenoside Rg3, (I) 20(R)-ginsenoside Rg3.

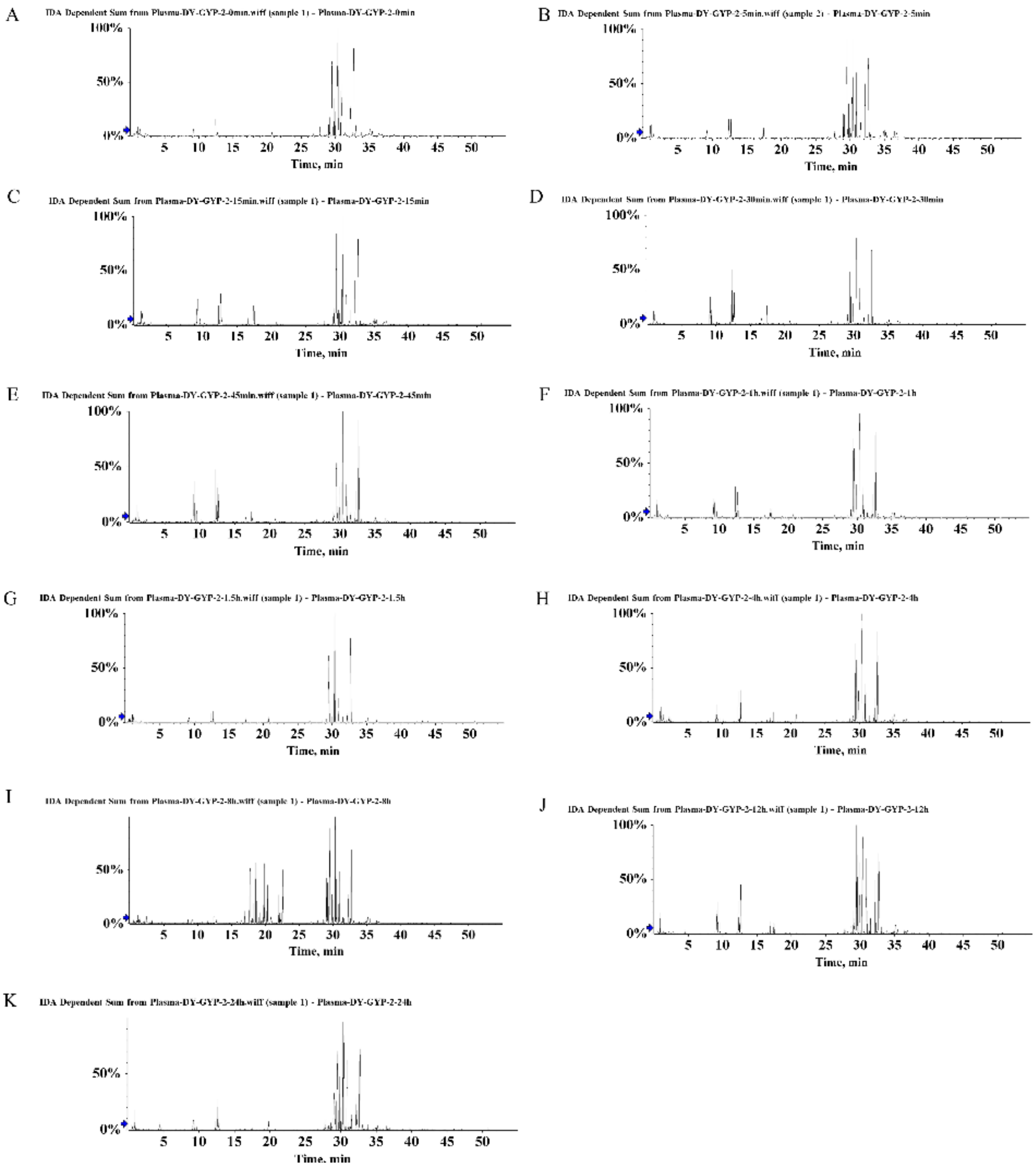

Figure S4. Thermal treatment of the ethanol extract of *Gynostemma pentaphyllum* and its blood component TIC diagram. (A) 0 min, (B) 5 min, (C) 30 min, (D) 45 min, (E) 1 h, (F) 1.5 h, (G) 4 h, (H) 8 h, (I) 12 h, (J) 24 h.

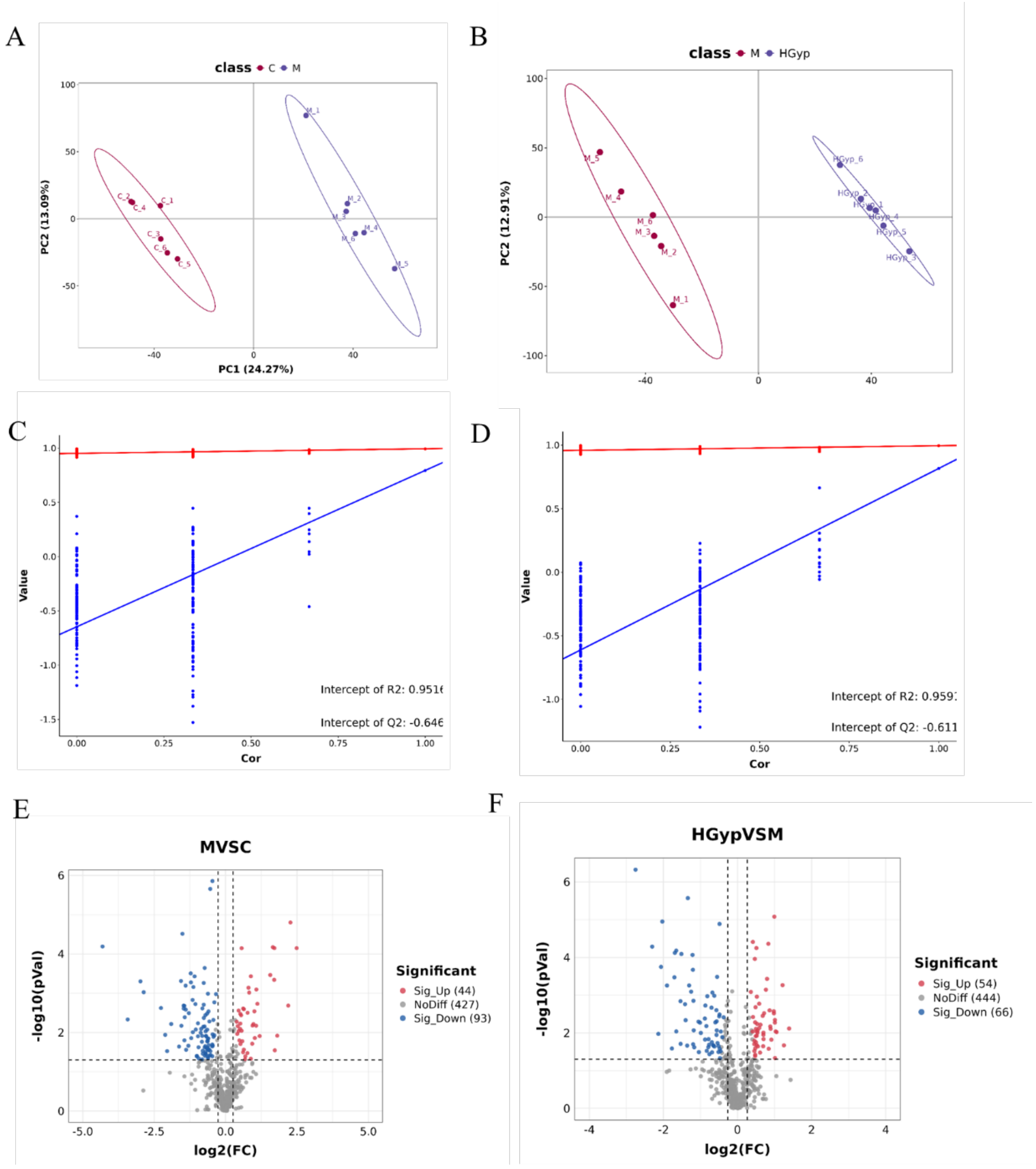

Figure S5. The influence of HGyp on the metabolites in mouse serum. (A)OPLS-DA comparison between the control and APAP group. (B) OPLS-DA comparison between the HGyp and APAP group. (C) Scatter Plot of the control vs. APAP group. (D) Scatter Plot of the HGyp vs. APAP group. (E) Volcano plots comparing the control with APAP group. (F) Volcano plot comparing the HGyp with APAP group. (Control group: C/SC; APPA group: M/SM; HGyp group: HGyp.)

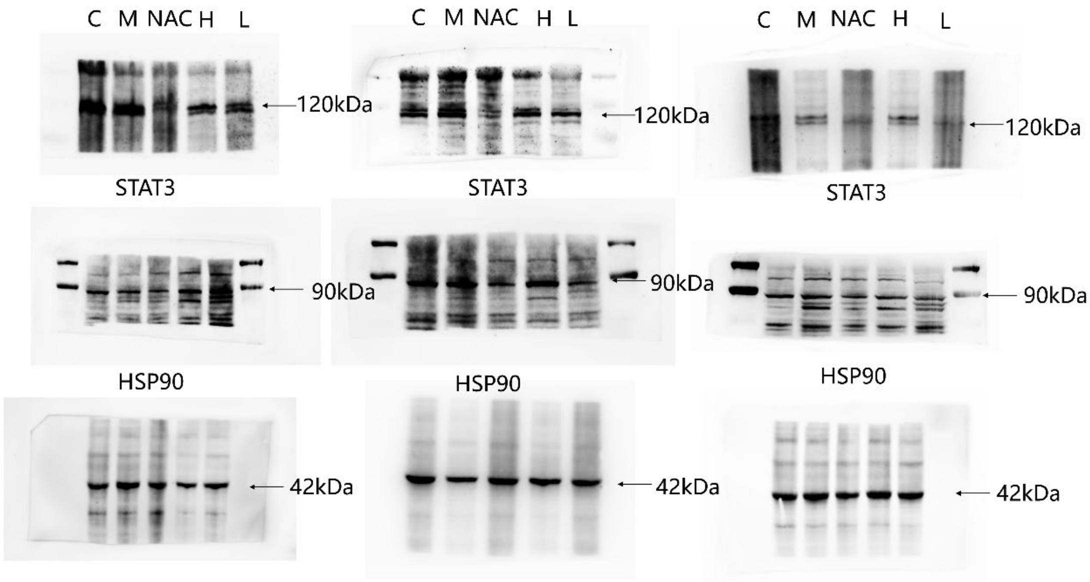

Figure S6. Effect of HGyp on the STAT3 and HSP90; original uncropped blots. (Control group: C; APAP group: M; NAC group: NAC; HGyp-H group: H; HGyp-L group: L.)

Table S1. Gypenosides identified by LC-MS.

NO.	RT/min	Compound	Molecular formula	$[M\text{-}H]^-$	fragments
1	7.172	gypenoside LVI	$C_{55}H_{90}O_{23}$	1093.6402	799.5357
2	8.453	gypenoside XLVI	$C_{48}H_{82}O_{19}$	961.5937	637.4629, 799.5282
3	9.097	Ginsenoside Rd	$C_{48}H_{82}O_{18}$	945.5987	765.5182, 621.4595
4	11.231	gypenoside L	$C_{42}H_{72}O_{14}$	799.5311	637.4680, 475.4032
5	11.518	gypenoside LI	$C_{42}H_{72}O_{14}$	799.5290	637.4638
6	11.672	20(S)-ginsenoside Rg3	$C_{42}H_{72}O_{13}$	783.5328	621.4662
7	11.938	20(R)-ginsenoside Rg3	$C_{42}H_{72}O_{13}$	783.5357	621.4718, 459.4072
8	12.395	damulin B	$C_{42}H_{70}O_{13}$	781.5180	619.4535
9	13.150	damulin A	$C_{42}H_{70}O_{13}$	781.4607	457.3549

Identify information on 9 compounds through HGyp.

Table S2. The differential metabolites in the serum of rats.

Compound	t_R/min	Molecular formula	$[M-H]^-$ (m/z)	Error (ppm)	Fragment ions (m/z)	Matabolicgeneration	Identification
M1	3.168	$C_{27}H_{30}O_{15}$	593.1520	1.4	429.0797、285.0400、284.0326、255.0288、227.0357、179.0021	prototype	quercetin-O-Hex-O-Pent
M2*	4.290	$C_{21}H_{18}O_{12}$	461.0731	1.2	285.0408、255.0307、227.0344、213.0552、185.0608、163.0026、113.0240、85.0287	phase II	Keampferol-3-glucuronide
M3	4.863	$C_{30}H_{26}O_{13}$	593.1316	2.6	447.0913、285.0398、284.0314、255.0289	prototype	kaempferol-O-neohesperidoside
M4	5.301	$C_{23}H_{30}O_{18}$	593.1340	-3.3	447.1000、285.0420、284.0348、255.0301	prototype	kaempferol-O-neohesperidoside
M5	8.206	$C_{22}H_{20}O_{13}$	491.0824	-1.5	315.0508、300.0273	phase II	Isorhamnetin-Glucuronic acid
M6	8.508	$C_{28}H_{32}O_{15}$	607.1660	-1.4	487.1274、461.1063、443.0965、299.0544、298.0474、283.0239、255.0286	prototype	methyl-kaempferol-O-neohesperidoside
M7	9.703	$C_{22}H_{20}O_{12}$	475.0875	-5.5	299.0566、284.0325、271.0621、255.0299、227.0351、175.0247、161.9955	phase II	methyl-kaempferol-Glucuronic acid
M8	9.853	$C_{22}H_{20}O_{12}$	475.0900	4.0	299.0573、284.0332、271.0616、255.0308、227.0351、175.0246、161.9962、113.0248	phase II	methyl-kaempferol-Glucuronic acid
M9	11.769	$C_{16}H_{12}O_9S$	379.0136	-4.8	299.0580、284.0329、271.0630	phase II	methyl-kaempferol-Sulfonation

Compound	t_R/min	Molecular formula	$[M-H]^-$ (m/z)	Error (ppm)	Fragment ions (m/z)	Matabolicgeneration	Identification
M10*	15.285	$C_{54}H_{92}O_{23}$	1107.6026	-7.5	N.F.	prototype	ginsenoside Rb1
M11*	15.638	$C_{53}H_{90}O_{23}$	1093.5928	1.7	931.5320、799.5014、637.1771、475.3820	prototype	gypenoside LVI
M12*	16.769	$C_{48}H_{82}O_{19}$	961.5404	2.8	799.4815、637.4313	prototype	gypenoside XLVI
M13	17.128	$C_{53}H_{90}O_{22}$	1077.5938	5.1	945.5769	prototype	ginsenoside Rb3
M14	17.560	$C_{54}H_{92}O_{22}$	1091.6102	4.7	945.5496、929.5530、783.4939、765.4827	prototype	gypenoside V
M15*	18.436	$C_{48}H_{82}O_{18}$	945.5480	4.5	783.4917、765.4847、621.4377、459.3854	prototype	ginsenoside Rd
M16	19.415	$C_{36}H_{60}O_{11}$	667.4046	-2.5	N.F.	phase I & II	Aglycone-Oxidation-Glucuronic acid
M17	19.763	$C_{30}H_{52}O_{6}$	507.3697	1.2	N.F.	phase I	Aglycone-Oxidation-Oxidation
M18	20.590	$C_{36}H_{60}O_{11}$	667.4090	4.1	N.F.	phase I & II	Aglycone-Oxidation-Glucuronic acid
M19	21.271	$C_{50}H_{84}O_{19}$	987.5593	5.0	945.5477、927.5453、765.4814	prototype	acetyl-ginsenoside Rd
M20	22.046	$C_{36}H_{60}O_{11}$	667.4092	4.4	473.3637	phase I & II	Aglycone-Oxidation-Glucuronic acid
M21	22.408	$C_{48}H_{82}O_{17}$	929.5506	2.9	783.4826、621.4426	phase I & II	Aglycone-Oxidation-Glucuronic acid
M22	22.669	$C_{36}H_{60}O_{10}$	651.4161	5.3	N.F.	phase I & II	Aglycone-Oxidation-Glucuronic acid
M23*	23.704	$C_{42}H_{72}O_{14}$	799.4891	5.2	637.4311、475.3802	prototype	gypenoside L
M24*	24.242	$C_{42}H_{72}O_{14}$	799.4884	4.3	637.4362	prototype	gypenoside LI
M25	24.311	$C_{36}H_{60}O_{10}$	651.4138	3.7	N.F.	phase I & II	Aglycone-Oxidation-Glucuronic acid
M26	24.625	$C_{36}H_{60}O_{10}$	651.4168	4.3	N.F.	phase I & e II	Aglycone-Oxidation-Glucuronic acid
M27	24.897	$C_{30}H_{52}O_{6}$	507.3688	-0.6	489.3604	phase I	Aglycone-Oxidation-Oxidation
M28	26.032	$C_{30}H_{52}O_{6}$	507.3692	0.2	489.3574	phase I	Aglycone-Oxidation-Oxidation

Compound	t_R/min	Molecular formula	$[M-H]^-$ (m/z)	Error (ppm)	Fragment ions (m/z)	Matabolicgeneration	Identification
M29*	26.188	$C_{42}H_{72}O_{13}$	783.4920	2.5	621.4356、459.3842	prototype	20(S)ginsenoside Rg3
M30*	26.577	$C_{42}H_{72}O_{13}$	783.4933	4.2	621.4398	prototype	20(R)ginsenoside Rg3
M31	26.668	$C_{44}H_{74}O_{15}$	841.5068	2.3	799.4920、637.4319	prototype	acetyl-gypenoside L
M32	26.944	$C_{44}H_{74}O_{15}$	841.5189	-3.2	799.4907	prototype	acetyl-gypenoside LI
M33*	27.777	$C_{42}H_{70}O_{13}$	781.4801	4.0	619.4230	prototype	damulin B
M34*	28.042	$C_{42}H_{70}O_{13}$	781.4825	-5.6	619.4219、457.3287	prototype	damulin A
M35	28.090	$C_{30}H_{52}O_5$	491.3761	3.9	473.3660	phase I	Aglycone-Oxidation-Oxidation
M36	28.671	$C_{30}H_{52}O_5$	491.3753	2.2	473.3616	phase I	Aglycone-Oxidation-Oxidation
M37*	29.290	$C_{42}H_{70}O_{12}$	765.4807	1.6	603.4310	prototype	ginsenoside Rk1
M38*	29.564	$C_{42}H_{70}O_{12}$	765.4829	1.8	N.F.	prototype	ginsenoside Rg5

* Confirmation of compound reference substances.

Table S3. Targeted analysis of water-soluble and medium-polarity metabolites using UHPLC-QE MS identified key differential metabolites.

No.	Metabolites	Metabolic pathway	RT min	Formula	VIP value	[a]fold change	[b]P-value
1	Palmitoyl Ethanolamide	Neuroactive ligand-receptor interaction	5.54	$C_{18}H_{37}NO_2$	1.19	1.33	0
2	Mannitol	Fructose and mannose metabolism	6.13	$C_6H_{14}O_6$	1.27	0.67	0
3	Cis-8,11,14-Eicosatrienoic acid	Linoleic acid metabolism	6.68	$C_{20}H_{34}O_2$	1.02	1.39	0.04
4	Laurate	Linoleic acid metabolism	4.57	$C_{12}H_{24}O_2$	1.31	1.62	0.01
5	Adrenic Acid	Fatty acid biosynthesis	6.77	$C_{22}H_{36}O_2$	1.42	1.6	0
6	Daidzein	Ferroptosis	2.45	$C_{15}H_{10}O_4$	1.78	0.5	0.01
7	Kynurenic acid	Biosynthesis of phenylpropanoids	1.86	$C_{10}H_7NO_3$	2.09	0.39	0.02
8	Adenosine	Sphingolipid signaling pathway	1.75	$C_{10}H_{13}N_5O_4$	1.57	0.55	0
9	Pantothenic acid	Pantothenate and CoA biosynthesis	1.75	$C_9H_{17}NO_5$	1.24	0.68	0
10	Dehydroascorbic acid	Ascorbate and aldarate metabolism	1.13	$C_6H_6O_6$	1.43	0.54	0.02
11	Docosahexaenoic acid	Biosynthesis of unsaturated fatty acids	5.72	$C_{22}H_{32}O_2$	1.4	1.63	0.01
12	Gingerol	Stilbenoid, diarylheptanoid and gingerol biosynthesis	3.42	$C_{17}H_{26}O_4$	2.33	0.31	0
13	4-Pyridoxic acid	Vitamin B6 metabolism	1.71	$C_8H_9NO_4$	1.49	0.49	0.03
14	D-Histidine	D-Amino acid metabolism	0.73	$C_6H_9N_3O_2$	1.43	0.62	0
15	Glutaric acid	Fatty acid degradation	0.99	$C_5H_8O_4$	1.37	0.65	0
16	trans-Cinnamic acid	Phenylpropanoid biosynthesis	2.04	$C_9H_8O_2$	1.49	0.56	0.01
17	Dodecanoic acid	Fatty acid biosynthesis	4.57	$C_{12}H_{24}O_2$	1.32	1.62	0.01
18	Chavicol	Phenylpropanoid biosynthesis	2.27	$C_9H_{10}O$	1.98	0.32	0.01
19	13-HODE	PPAR signaling pathway	5.5	$C_{18}H_{32}O_3$	1.39	1.62	0.02
20	Bergapten	Biosynthesis of phenylpropanoids	0.79	$C_{12}H_8O_4$	1.08	0.7	0.02
21	Cinnamaldehyde	Inflammatory mediator regulation of TRP channels	2.08	C_9H_8O	1.99	0.43	0

[a] Only metabolites with variable influence on projection (VIP) values of greater than 1.0 and *p*-values of less than 0.05 were deemed statistically significant.

[b] Fold change was calculated as the logarithm of the average mass response (area) ratio between the two classes (i.e., Fold change = lg [HGyp/model]).

Table S4. Analysis of the entire metabolome detected by UHPLC-QE MS revealed key differential metabolites.

No.	Metabolites	Metabolic pathway	RT min	Formula	VIP value	[a]fold change	[b]P-value
1	1-heptadecanoyl-sn-glycero-3-phosphocholine	Glycerophospholipid metabolism	4.51	$C_{25}H_{52}NO_7P$	1.31	0.65	0.01
2	Laurate	Fatty acid biosynthesis	4.57	$C_{12}H_{24}O_2$	2.10	0.36	0.002
3	3-phenyllactic acid	Tropane, piperidine and pyridine alkaloid biosynthesis	1.96	$C_9H_{10}O_3$	1.19	1.53	0.03
4	Adrenic Acid	Biosynthesis of unsaturated fatty acids	6.77	$C_{22}H_{36}O_2$	1.12	0.73	0.02
5	Daidzein	Metabolic pathways	2.45	$C_{15}H_{10}O_4$	1.27	1.44	0.003
6	Adenosine	Metabolic pathways	1.75	$C_{10}H_{13}N_5O_4$	1.23	1.50	0.02
7	Pantothenic acid	Metabolic pathways	1.75	$C_9H_{17}NO_5$	1.20	1.43	0.01
8	Gingerol	Stilbenoid, diarylheptanoid and gingerol biosynthesis	3.42	$C_{17}H_{26}O_4$	2.27	0.96	0.0003
9	LPC 18:3	Glycerophospholipid metabolism	3.45	$C_{26}H_{48}NO_7P$	1.71	0.57	$3.6*10^{-6}$
10	PC(18:2/0:0)	Glycerophospholipid metabolism	3.43	$C_{26}H_{51}N_1O_7P_1$	1.62	0.58	0.00081
11	PE(20:3/0:0)	Glycosylphosphatidylinositol (GPI)-anchor biosynthesis	3.45	$C_{25}H_{45}N_1O_7P_1$	2.21	0.38	0.000082
12	D-Histidine	D-Amino acid metabolism	0.73	$C_6H_9N_3O_2$	1.40	1.56	0.0025
13	trans-Cinnamic acid	Phenylalanine metabolism	2.04	C9H8O2	1.68	1.93	0.0064
14	LysoPC(18:3(6Z,9Z,12Z)/0:0)	Glycerophospholipid metabolism	3.43	$C_{26}H_{48}NO_7P$	1.69	0.59	$2.61*10^{-6}$
15	LPC 19:0-SN1	Glycerophospholipid metabolism	5.78	$C_{27}H_{56}NO_7P$	1.27	0.67	0.01
16	Dodecanoic acid	Fatty acid biosynthesis	4.57	$C_{12}H_{24}O_2$	2.08	0.37	0.0020
17	LysoPE(20:3(11Z,14Z,17Z)/0:0)	Glycerophospholipid metabolism	3.97	$C_{25}H_{46}NO_7P$	1.30	1.47	0.0058
18	Sphingosine-1-phosphate	Sphingolipid metabolism	3.94	$C_{18}H_{38}NO_5P$	1.13	1.32	0.001

19	LPI 18:2	Glycerophospholipid metabolism	2.87	$C_{27}H_{49}O_{12}P$	1.03	0.78	0.01
20	13-HODE	Linoleic acid metabolism	5.50	$C_{18}H_{32}O_3$	1.62	0.55	0.010
21	LPC 22:5	Glycerophospholipid metabolism	4.10	$C_{30}H_{52}NO_7P$	1.21	0.64	0.030
22	Cis-11,14-Eicosadienoic acid	Biosynthesis of unsaturated fatty acids	7.16	$C_{20}H_{36}O_2$	1.08	0.73	0.020
23	11-eicosenoic acid	Biosynthesis of unsaturated fatty acids	8.03	$C_{20}H_{38}O_2$	1.26	0.64	0.025
24	Cinnamaldehyde	Metabolic pathways	2.08	C_9H_8O	1.60	1.75	0.00067
25	Cis-11-Eicosenoic acid	Biosynthesis of unsaturated fatty acids	8.03	$C_{20}H_{38}O_2$	1.24	0.65	0.025

[a] Only metabolites with variable influence on projection (VIP) values of greater than 1.0 and *p*-values of less than 0.05 were deemed statistically significant.

[b] Fold change was calculated as the logarithm of the average mass response (area) ratio between the two classes (i.e., Fold change = lg [HGyp/model]).

Table S5. Binding energy of gypenosides based on the Gold docking simulation (kcal/mol)

Protein		AKT1	MMP2	GRB2	FGF2	STAT3	CASP3	HSP90
Damulin A	Fitness	20.94	27.90	0	35.14	38.09	30.52	41.94
	S(hb_ext)	0.20	0	3.60	5.56	7.93	0.11	1.36
	S(vdw_ext)	15.83	30.4	-1007.07	28.85	23.74	22.95	31.27
	S(hb_int)	0	0	0	0	0	0	0
	S(int)	-1.02	-13.9	-77.03	-10.09	-2.48	-1.14	-2.41
	intcor	-496.02	-512.84	-525.17	-496.15	-497.62	-508.34	-514.59
Damulin B	Fitness	27.86	41.15	0	24.43	40.29	42.98	54.46
	S(hb_ext)	6.68	3.97	10.55	3.36	2.49	13.10	1.88
	S(vdw_ext)	16.61	27.85	-182.15	15.68	27.92	34.30	39.61
	S(hb_int)	0	0	0	0	0	0	0
	S(int)	-1.66	-1.11	-157.82	-0.48	-0.59	-17.29	-1.89
	intcor	-492.09	-493.96	-509.60	-483.48	-488.58	-496.17	-499.99
Gypenoside L	Fitness	0.52	46.14	-346.26	37.87	57.07	59.96	49.43
	S(hb_ext)	0.45	8.27	24.00	7.18	15.88	8.33	0.36
	S(vdw_ext)	7.54	29.37	-253.19	28.58	30.82	38.57	39.07
	S(hb_int)	0	0	0	0	0	0	0
	S(int)	-10.29	-2.52	-22.12	-8.61	-1.19	-1.40	-4.66
	intcor	-41.35	-31.9	-40.96	-30.07	-30.23	-31.91	-30.94
Gypenoside LI	Fitness	32.64	14.81	0	44.97	38.83	32.97	45.21
	S(hb_ext)	3.47	3.03	3.56	4.58	10.54	2.00	6.35
	S(vdw_ext)	21.65	13.25	-1781.42	35.04	26.26	25.54	31.24
	S(hb_int)	0	0	0	0	0	0	0
	S(int)	-0.59	-6.45	-4.49	-7.80	-7.81	-4.15	-4.09
	intcor	-39.66	-37.14	-44.62	-31.05	-28.73	-37.40	-36.31
Gypenoside LVI	Fitness	2.31	0	0	43.22	37.42	0	51.95
	S(hb_ext)	0	4.25	5.90	8.70	6.26	9.41	9.37
	S(vdw_ext)	7.55	-8.33	-63.73	25.77	23.73	-57.77	43.73
	S(hb_int)	0	0	0	0	0	0	0
	S(int)	-8.07	-606.00	-421.38	-0.91	-1.47	-754.76	-17.55
	intcor	-92.2	-51.68	-47.39	-41.21	-37.48	-59.54	-38.56
Gypenoside	Fitness	17.2	30.98	0	51.19	46.51	7.77	46.90

XLVI								
	S(hb_ext)	6.69	8.59	14.74	9.94	8.39	0	12.61
	S(vdw_ext)	18.25	25.40	-75.79	35.28	30.32	15.09	26.47
	S(hb_int)	0	0	0	0	0	0	0
	S(int)	-14.58	-12.53	-227.46	-7.27	-3.57	-12.98	-2.12
	intcor	-57.01	-45.59	-51.12	-40.29	-39.79	-45.65	-32.99
Gypenoside Rd	Fitness	27.63	30.58	-230.28	35.57	37.77	38.83	27.92
	S(hb_ext)	3.20	1.92	1.44	2.91	4.44	0	4.31
	S(vdw_ext)	18.30	20.86	-156.98	23.63	26.46	29.90	18.77
	S(hb_int)	0	0	0	0	0	0	0
	S(int)	-0.74	-0.02	-15.88	-0.84	-3.04	-2.28	-2.20
	intcor	-19.81	-16.47	-24.80	-17.65	-17.76	-18.77	--17.68
20R ginsenoside Rg3	Fitness	12.10	46.22	0	43.52	42.13	-164.05	45.89
	S(hb_ext)	3.41	8.27	3.61	14.37	1.76	8.68	1.53
	S(vdw_ext)	10.12	30.90	-255.01	30.93	31.68	-105.62	37.92
	S(hb_int)	0	0	0	0	0	0	0
	S(int)	-5.21	-4.54	-125.79	-13.37	-3.20	-28.05	-7.79
	intcor	-32.66	-26.88	-40.27	-27.99	-27.22	-36.12	-32.22
20S ginsenoside Rg3	Fitness	0	0	-312.99	40.97	41.61	40.81	49.99
	S(hb_ext)	0.94	2.44	6.67	13.86	5.21	6.29	3.89
	S(vdw_ext)	-117.44	- 1429.24	-190.06	20.41	30.01	33.00	37.25
	S(hb_int)	0	0	0	0	0	0	0
	S(int)	-304.64	-46.53	-58.33	-0.96	-4.87	-10.85	-5.12
	intcor	-46.73	-36.10	-37.62	-25.28	-24.90	-29.38	-29.16

The molecular docking results for 9 compounds, including Fitness, S(hb_ext), S(vdw_ext), S(hb_int), S(int), and intcor.
